# A Conversational Multi-Agent AI System for Integrated Multi-Omics Analysis and Biomedical Discovery

**DOI:** 10.64898/2026.08.08.743577

**Published:** 2026-08-14

**Authors:** Pankaj Rajdeo, Shunya Asanuma, Michal Kouril, Peixin Lu, Jichao Chen, Aman Chadha, V. B. Surya Prasath, Bruce J. Aronow, Nathan Salomonis

**Affiliations:** 1 Division of Biomedical Informatics, Cincinnati Children’s Hospital Medical Center, Cincinnati, OH, USA.; 2 Department of Biomedical Informatics, University of Cincinnati, OH, 45267 USA.; 3 Department of Pediatrics, College of Medicine, University of Cincinnati, OH, 45267 USA.; 4 Division of Pulmonary Biology, Cincinnati Children’s Hospital Medical Center, Cincinnati, OH, 45229 USA.; 5 Apple Corporation, Cupertino, CA 95014, USA.; 6 Department of Computer Science, University of Cincinnati, Cincinnati, OH 45229, USA.

## Abstract

Single-cell and spatial omics offer unprecedented opportunities to decipher the mechanisms of disease, however, this process requires teams of experts, iterative trial-and-error and reasoning across modalities. Here we present LungChat (https://chat.lungmap.net), a conversational system for integrated multi-omics analysis and biomedical discovery, deployed as a hierarchical multi-agent architecture in which a supervisor decomposes natural-language questions into parallel, tool-grounded tasks spanning single-cell and spatial analyses, literature and clinical-trial synthesis, and drug repurposing. To predict new therapeutics, LungChat implements Direction-Aware Repurposing and Targeting (DART) to distinguish perturbations that reverse disease transcriptional programs from those that reinforce them, at the cell-type level, for safety prediction. Controlled architecture ablations showed that hierarchical orchestration improved grounded abstention and token efficiency and preserved strong performance on complex multi-step tasks. In pulmonary disease case studies, LungChat independently prioritized saracatinib for IPF through drug-connectivity screening, followed by DART-based cell-type analysis; the same compound has been evaluated in the STOP-IPF clinical trial (NCT04598919). The system also recovered fluticasone propionate, an established COPD therapy, through a single orchestrated analysis. This tissue-agnostic system provides a blueprint for verifiable agentic AI systems that support reproducible scientific discovery.

## 1 Introduction

Advances in single-cell and spatial transcriptomics have fundamentally changed how biological systems are studied, enabling cell-type–resolved and spatially contextualized analyses across development, homeostasis, and disease. Large-scale atlas efforts such as The Human BioMolecular Atlas Program (HuBMAP) [4], Human Cell Atlas (HCA) [21, 22], and LungMAP [6] have generated millions of profiles across the spectrum of human tissues, development and disease, creating unprecedented opportunities for systems-level discovery. However, the increased scale and complexity of these datasets, shifts the challenge from analysis to systems-level integration. This challenge necessitates integration of insights across omics technologies, while reconciling inconsistent metadata across studies.

Existing solutions address parts of this problem but remain structurally limited. Interactive visualization platforms such as the CZI CellxGene, and UCSC cell browsers [20, 26] enable exploration of atlas-level datasets but lack support for deep, quantitative analysis. General-purpose AI assistants (e.g., ChatGPT, Claude, etc) can synthesize vast amounts of prior knowledge from the literature but lack harmonized quantitative data, metadata or access to validated analysis tools, resulting in “best guesses” [12]. As a result, there is a growing disconnect between the availability of atlas-scale data and the ability of researchers to interrogate it rigorously and iteratively.

Recent progress in large language models (LLMs) and agentic AI systems suggests a path forward. Conversational interfaces have demonstrated promise for lowering the barrier to complex analyses [23]. Pretrained language models and single-agent approaches, such as CellWhisperer [23], CompBioAgent [29], Cell2Text [13] and scChat [17] generate rapid and automated annotation and visualization of single-cell genomics data. Multi-agent architectures such as BioMaster [27], BioAgents [18] and Biomni [10] enable inter-agent coordination, planning and task execution to nominate hypotheses for validation. Existing approaches such as DrugAgent [11] and TxGNN [9], provide exciting new approaches to augment expert-driven drug repositioning. Despite these innovations, numerous challenges remain in AI assisted drug discovery and biomedical reasoning, including standardized integration of findings across studies and modalities (space, time, and disease); functional prediction (pathways, regulatory potential, and cellular interactions); assessment of the potential positive and negative impacts of new therapies; and contrasting findings across disease and developmental contexts.

In this work, we introduce LungChat, a conversational multi-agent AI system for integrated multi-omics analysis, designed to support transparent, reproducible biomedical discovery through natural language interactions. LungChat is open-access at https://chat.lungmap.net and leverages 39 single-cell datasets spanning 2.8 million cells, integrated spatial omics technologies and over 50 analytical tools. The system combines (i) **multi-agent orchestration**, in which a supervisor coordinates specialized sub-agents; (ii) **tool-grounded analysis**, grounding language model outputs in structured execution of validated bioinformatics methods including functional enrichment (ToppGene [5]), drug connectivity and repurposing (iLINCS [19]), literature retrieval and synthesis (PubMed); and (iii) **provenance-tracked reproducibility**, where every analytical step produces persistent, publication-ready outputs with complete parameter provenance. For therapeutic hypothesis generation, we developed Direction-Aware Repurposing and Targeting (DART), a method for cell-type-resolved drug prioritization that classifies perturbations as repression or activation to predict on-target efficacy and off-target safety at cell-type resolution (Section 4.7). Applied to idiopathic pulmonary fibrosis (IPF), LungChat independently prioritized saracatinib through drug-connectivity screening, followed by DART-based cell-type analysis; the same compound has been evaluated in the STOP-IPF clinical trial (NCT04598919). In Chronic Obstructive Pulmonary Disease (COPD), which affects over 300 million individuals, DART identifies fluticasone propionate, a corticosteroid used in COPD combination therapy, among the top predicted disease-reversal signatures. By aligning natural language interaction with real analytical execution, this work positions conversational multi-agent systems as a practical interface for atlas-scale data exploration. The system complements emerging AI scientist approaches [7, 16] by prioritizing transparent, tool-grounded analysis over autonomous hypothesis generation, addressing a critical gap between accessibility and rigor in contemporary biomedical research.

## 2 Results

### 2.1 Coordinated multi-agent analysis across omics and biomedical knowledge

LungChat was developed to translate complex biomedical questions into structured analyses by leveraging harmonized omics datasets, reproducible analytical results, publication-ready visualizations, and literature grounded evidence synthesis. Where possible, computationally intensive analyses are precomputed, enabling rapid retrieval of results from harmonized datasets and metadata. Each analytical step is grounded in the structured execution of validated bioinformatics methods and produces persistent, publication-ready outputs with complete parameter provenance, allowing analyses to be inspected, reproduced, and extended. Rather than generating plausible but unverified responses, LungChat returns evidence-grounded results that researchers can examine and rerun.

LungChat implements a hierarchical multi-agent architecture with a central stateful supervisor agent that coordinates the analysis and maintains the conversational state that serves as the system’s working memory (Figure 1). This statefulness enables multi-turn, iterative dialogue in natural language. The supervisor coordinates a team of specialized subagents, each augmented with a library of validated analytical tools: a single-cell agent for differential expression, cell-type characterization, correlation, and visualization; a spatial agent for spatial gene-expression mapping, cell-cell communication inference, and neighborhood enrichment within and across technologies; a research agent that bridges internal data with external knowledge through functional enrichment, literature synthesis, clinical-trial retrieval, drug connectivity, and repurposing; and a LungMAP agent for natural-language exploration of the LungMAP consortium database. For analyses not covered by the core tools, the supervisor can generate and execute custom code in a secure sandbox. Functional enrichment is performed with ToppGene, drug connectivity and repurposing through iLINCS, and literature synthesis across EuropePMC. User queries are decomposed into tractable subtasks that execute in parallel when independent, and their results are synthesized into coherent scientific responses. To increase analytical throughput, the supervisor can invoke multiple subagents concurrently, including multiple instances of the same subagent when appropriate.

For therapeutic hypothesis generation, the research agent’s drug connectivity and repurposing analysis is coupled to Direction-Aware Repurposing and Targeting (DART), a method for cell-type-resolved drug prioritization that classifies perturbations as reversing or reinforcing a disease transcriptional program to predict on-target efficacy and off-target safety at cell-type resolution.

### 2.2 Benchmarking and architecture ablation

We evaluated LungChat using a comprehensive benchmark comprising 100 test cases across five evaluation tracks: 50 single-step analytical queries (analytical workflows, data visualization, metadata and knowledge queries), 13 security and adversarial queries (prompt injection, security exploitation, out-of-domain), 17 grounded abstention queries testing appropriate scope limitation, 10 multi-tool orchestration queries requiring cross-domain coordination, and 10 decomposition-stress queries testing scientific reasoning depth. To assess the contribution of the multi-agent architecture, we conducted ablation experiments across four configurations. Following current evaluation best practices [3], we employed both deterministic code-based evaluators (configuration correctness, execution success, metadata consistency, tool selection accuracy) and LLM-as-judge scoring using GPT-4o (temperature=0) with a structured binary rubric covering biological accuracy, visual correctness, and refusal appropriateness. Each test case was executed across three independent trials (*n*=3 repetitions per case) to assess reliability.

To isolate the contribution of hierarchical multi-agent orchestration, we evaluated LungChat under four ablation configurations: (1) **MA+Sel** (production), the hierarchical architecture with hybrid tool selection; (2) MA−Sel, the production hierarchy without the tool selector; (3) Flat+Sel, a single agent with all 51 tools and merged domain prompts, retaining the tool selector; and (4) **Flat**−**Sel**, a single agent with all tools, merged prompts, and no selector. Flat-agent baselines preserve all domain knowledge from specialist prompts, ensuring differences reflect architectural design rather than information asymmetry.

Figure 2 summarizes the ablation results across all five tracks (100 questions, 1,080 total evaluations). On single-step queries (Figure 2a), all four configurations achieved ≥89% LLM Judge accuracy, with Flat−Sel scoring highest (98.0%), confirming that single-step quality is architecture-neutral. The hierarchical architecture achieves a consistent ~2× reduction in token use on single-step queries and other lower-complexity evaluation tracks (Figure 2b), reflecting the context-partitioning benefit of scoped tool pools described in Section 4.4.2. Grounded abstention (Figure 2c) revealed the largest architectural difference: MA+Sel blocked 90.2% of disallowed tool invocations, compared with 54.9% for Flat−Sel, a difference of 35.3 percentage points. On complex multi-tool and decomposition-stress queries (Figure 2d), both architectures achieve comparable quality, confirming that analytical depth stems from domain engineering rather than hierarchy. Full per-track results are provided in Appendix F.

Across the tested adversarial tasks, the system prevented all prompt-injection, developer-mode, and instruction-override attempts, as well as all attempts to perform system reconnaissance or extract environment variables. All analytical outputs are saved with complete parameter provenance to support reproducible re-execution (Appendix C).

### 2.3 Cell-type-resolved therapeutic prioritization and mechanistic exploration

To evaluate LungChat’s ability to plan and execute multi-step workflows and synthesize their results, we focused on pulmonary fibrosis, for which effective therapeutic options remain limited. Existing therapies have largely aimed to suppress the activation, migration, and proliferation of resident lung fibroblasts, processes that contribute to tissue scarring and impaired lung function.

As a first evaluation, we explicitly asked LungChat to identify therapeutic targets for pulmonary fibrosis based on differential expression, pathway analysis, spatial location, and cell communication analyses. Our supervisor agent decomposed this query into six coordinated subtasks, executing independent analyses in parallel and dependent analyses sequentially (Figure 3a,b).

These analyses successfully identify well-defined pulmonary fibrosis marker genes (e.g., IL13RA2, COL1A1) and enriched pathways of IPF (e.g., extracellular matrix deposition, P53 transcriptional regulation, VEGFA and TGF-*β* signaling; Figure 3c–e). Integrated spatial-based cell-communication analyses (Squidpy, CellChat) further nominated EREG and VEGFA signaling from aberrant basaloid cells (pulmonary fibrosis KRT5−/KRT17+ alveolar type 2 cells) to activated fibroblasts, as uniquely spatial enriched interactions in pulmonary fibrosis versus controls (Figure 3f). This result is supported by VEGFA/VEGFR2 signaling from unbiased global gene set enrichment (Figure 3d). LungChat performed drug repurposing analysis via iLINCS connectivity mapping combined with DART cell-type safety profiling. In addition to recovering the approved IPF therapy nintedanib and the drug-associated target PDGFRB, LungChat identified saracatinib, a selective SRC kinase inhibitor, as the top therapeutic candidate (Figure 3g). DART cell-type impact analysis across 475 lung cellular signatures demonstrated that Saracatinib exhibits 90.9% reversal of pathogenic myofibroblast signatures (Pearson *r* = −0.82) and 87.5% reversal of alveolar fibroblast activation (*r* = −0.75), with an average fibroblast specificity of 54.8%. Off-target safety profiling identified potential risks in fetal/adult smooth muscle and pericytes, affecting approximately a dozen genes in each. Saracatinib had previously demonstrated superior anti-fibrotic efficacy compared to nintedanib and pirfenidone in preclinical models [1] and has been evaluated in STOP-IPF (NCT04598919), providing external support for the overall prioritization. This therapeutic target identification was performed as a single natural-language prompt submitted to a fresh LungChat session with no prior conversational context or iterative prompt refinement; the complete execution trace, including all tool calls, parameters, and intermediate outputs, is provided in Appendix E.

As a separate evaluation, we asked LungChat to broadly predict drugs that would reverse disease gene expression profiles in any (separate) or all (union) cell populations from patients with COPD. This analysis identified fluticasone propionate, an inhaled corticosteroid used in COPD combination therapy, as among the top predicted anti-COPD drug reversal signatures in dendritic cells. Fluticasone Propionate is a synthetic trifluorinated corticosteroid which reduces airway inflammation in moderate-to-severe COPD in concert with *β*2-agonists, such as Fenoterol. Fenoterol was identified among the top 3 anti-COPD drug reversal signatures, when all cell type COPD DEGs were aggregated (union) by LungChat. DART predicted off-target reversal of fetal and adult normal gene expression profiles in fetal smooth muscle, neonatal secondary crest myofibroblasts and adult ionocytes. Given the rarity of ionocytes and relative recent nature of their functional characterization, such cellular impacts would likely go unnoticed in conventional off-target screening cellular platforms.

#### 2.3.1 Regulatory mechanism evaluation

In addition to the discovery and safety evaluation of promising therapeutic candidates, LungChat can deeply evaluate regulatory mechanisms from a diverse repertoire of omics-focused analyses and the scientific literature. To assess this capability, LungChat was tasked to evaluate the potential mechanisms of action of a new drug target under current FDA evaluation. Lysophosphatidic acid receptor (LPAR) inhibitors target TGF-beta signaling pathway members, which are well-documented to be up-regulated in pulmonary fibrosis. To determine the mechanism of action of these inhibitors, LungChat executed a multi-step analysis to: 1) identify candidate drugs in iLINCS, 2) determine which cell types express LPAR1, 3) identify its spatial niches, 4) determine which cell-type disease programs could be reversed by drug treatment and to identify which transcriptional regulators were upstream of pharmacologically reversed genes (transcriptionally). These analyses confirmed expression of LPAR1 in all adult lung fibroblast and pericyte populations, confirmed spatial expression of LPAR1 with activated fibroblasts in the IPF niche, identified expected and novel IPF cellular impacts (reversal of IPF expression in fibroblasts and myeloid cells, respectively) and identified SMAD3 as the upstream transcriptional regulator of LPAR inhibitor targets genes (upregulation of SERPINE1). However, DART identified approximately a dozen potential off-target effects, including in presumably healthy smooth muscle, lymphatic endothelial, submucosal gland, deutrosomal, fetal Schwann cell and myofibroblasts with LPAR1 inhibition. Thus, LungChat is able to autonomously define complex regulatory mechanisms underlying drug treatment, including known and novel predicted interactions.

## 3 Discussion

We present LungChat, a conversational multi-agent AI system for integrated multi-omics analysis that supports transparent, end-to-end scientific workflows across single-cell and spatial transcriptomic data, along with pseudobulk expression summaries, while reducing the need for manual scripting. Architecture ablation across five evaluation tracks (1,080 evaluations) showed that the evaluated architectures achieved broadly comparable analytical quality on complex tasks, while the multi-agent hierarchy provided approximately 2× greater token efficiency and a 35-percentage-point improvement in grounded abstention. These results support the value of context partitioning and hierarchical orchestration for efficient and appropriately constrained analytical systems.

Through a series of therapeutically focused case studies, we demonstrate that LungChat can recover established disease-associated biology, characterize regulatory processes, compare diseased and healthy spatial niches, and identify known antifibrotic therapies. Beyond recapitulating known biology, we introduce Direction-Aware Repurposing and Targeting (DART), a cell-type-resolved method that assesses whether perturbation-induced transcriptional signatures reverse or reinforce disease-associated programs. Applied to IPF, LungChat independently prioritized saracatinib through drug-connectivity screening, followed by DART-based cell-type analysis; the same compound has been evaluated in the STOP-IPF clinical trial (NCT04598919), providing external support for the biological relevance of this prioritization. In COPD, DART recovered fluticasone propionate, an established therapy, supporting the ability of the method to identify clinically relevant perturbations from cell-type-resolved disease signatures.

More broadly, DART can compare disease-associated, developmental, and perturbation-induced transcriptional programs across cell types, datasets, and technologies. Such comparisons may reveal conserved biological programs, technology-associated differences, and potentially analogous or inconsistently annotated cell populations across studies. These analyses could support drug-discovery workflows by identifying molecular pathways, cell populations, and disease contexts predicted to be reversed or reinforced by a perturbation. By revealing heterogeneous perturbation effects across cell populations, DART supports efficacy-oriented hypothesis generation and highlights potential off-target effects that may be obscured in bulk analyses.

Importantly, LungChat emphasizes analytical correctness and reproducibility rather than fully autonomous hypothesis generation. Analytical steps produce provenance-tracked outputs that record the data, parameters, tools, and intermediate results required to inspect and rerun the underlying analyses. Although developed for lung biology, the architecture is tissue-agnostic, with modular agents and standardized data schemas that support extension to other organs and multi-omics domains. LungChat therefore provides an extensible system for integrated data analysis and evidence-grounded biological interpretation.

In summary, our system demonstrates how conversational multi-agent systems can serve as practical scientific interfaces that connect natural-language research questions with coordinated, reproducible computational analyses and experimentally testable hypotheses. By combining foundation models with validated analytical tools and provenance-tracked execution, LungChat provides a blueprint for transparent agentic AI systems designed to support, rather than replace, biological research.

### Use of large language models:

This manuscript was written by the authors with assistance from large language models for grammar improvement, sentence clarity, and writing refinement. All scientific content, results, analyses, and conclusions were developed by the authors, who take full responsibility for the accuracy and validity of all claims.

## 4 Methods

### 4.1 Problem formulation and scope

We formalize the task of interactive multi-omics analysis as follows. Given a natural language query q∈𝒬, a collection of multi-omics datasets $𝒟=\left\{D_{sc},D_{st},D_{pb}\right\}$ spanning single-cell RNA-seq, spatial transcriptomics, and pseudobulk expression data, and a library of analytical tools $𝒯=\left\{t_{1},\ldots ,t_{n}\right\}$, our goal is to generate a structured response ℛ=(𝒱,ℐ,𝒜) comprising visualizations 𝒱, biological insights ℐ, and reproducible outputs 𝒜.

The central challenge lies in decomposing unstructured scientific queries into executable analytical workflows while ensuring biological accuracy, cross-modal data integration, and full reproducibility. Unlike single-turn question answering, interactive analysis requires maintaining analytical state across multi-step investigations where subsequent queries build upon prior results.

### 4.2 System overview

LungChat is an AI system for integrated multi-omics analysis, designed to support biomedical discovery through interactive analysis. The system employs a hierarchical multi-agent architecture where a supervisor agent orchestrates specialized sub-agents, each augmented with domain-specific analytical tools (Figure 1). User queries are decomposed into tractable sub-tasks that execute in parallel when independent, with results synthesized into coherent scientific responses.

The architecture integrates three core capabilities: (1) multi-agent orchestration for complex query handling, (2) tool-augmented reasoning enabling precise analytical operations, and (3) unified access to heterogeneous omics data through ontology-grounded harmonization. Intermediate analytical outcomes serve as feedback signals that update the supervisor’s task decomposition, analytical resolution, and operator selection, establishing a computational lab-in-the-loop paradigm where data-driven results guide subsequent analytical actions without retraining or parameter updates.

### 4.3 Analytical workflows

#### 4.3.1 Omics datasets

LungChat operates over precompiled single-cell gene expression and spatial transcriptomics omics datasets assembled from > 37 studies curated by the LungMAP Consortium and the Human Cell Atlas initiative. Single-cell datasets are provided as CellxGene-curated .h5ad objects with consistent, standardized mappings to CellxGene-supported biomedical ontologies, including harmonized cell-type, disease, developmental stage, and assay annotations. To enable interactive analysis at scale, metacells and differential expression statistics are precomputed.

In addition to expression measurements, regulatory priors derived from ChIP-seq experiments and protein–protein interaction networks from prior studies are embedded locally within each .h5ad object using the NetPerspective software. This enables rapid retrieval of gene regulatory and interaction context during downstream analyses.

Spatial transcriptomics data include matched image-based (Xenium) and whole-transcriptome (Visium-HD) profiles. Integration between Xenium and Visium-HD datasets is performed through spot- and cell-segmented alignment and annotation stability that enables cross-platform comparison of spatial gene expression patterns and tissue organization across technologies. Appendix A provides details of the dataset curation and preprocessing undertaken.

#### 4.3.2 Specialized tools

Analytical workflows in LungChat are executed through a library of 51 specialized analytical and knowledge tools distributed across four specialist agents: the Single-Cell agent (13 tools for differential expression, visualization, and correlation analysis), Spatial agent (26 tools for spatial mapping, cell-cell communication, and neighborhood enrichment), Research agent (4 tools for functional enrichment, drug connectivity, literature search, and clinical trials), and Atlas Agent (8 tools for LungMAP/ontology access). Single-cell tools are implemented in Python using Scanpy, Matplotlib, Seaborn, and iGraph; spatial transcriptomics tools are implemented in R using Seurat, CellChat, and Squidpy. Table B1 (Appendix B) provides a complete reference for analytical, external API, and knowledge tools. At runtime these operators execute outside the language-model context as calls to dedicated containerized analysis services: single-cell tools are dispatched to a FastAPI Python sidecar (Scanpy, Matplotlib, Seaborn, iGraph) and spatial tools to an R Plumber sidecar (Seurat, CellChat, Squidpy), while user- or agent-authored custom analyses run in a separate code-execution sandbox that supports both Python and R (e.g., Seurat, tidyverse, Bioconductor) and executes as an unprivileged user. All services run as isolated Docker containers communicating over an internal bridge network authenticated by a service token, with per-request timeouts and thread/user path isolation, decoupling numerical computation from agent reasoning and from other users’ sessions.

All tools are accessed through a standardized JSON-based interface that specifies input datasets, parameter settings, and expected outputs. This abstraction enforces reproducibility and allows analytical steps to be executed deterministically. Visualization operators generate publication-ready figures in both raster (PNG) and vector (PDF) formats, accompanied by tabular outputs (TSV). For exploratory inspection of high-dimensional datasets, LungChat provides an interactive heatmap interface using Morpheus and dedicated applications for single-cell label projection (Azimuth), visualization (ShinyCell) and exploration of spatial omics (Vitessce).

#### 4.3.3 External knowledge and tools

LungChat integrates a set of API-backed external tools for functional enrichment, literature retrieval and synthesis, clinical trials, and drug connectivity analysis. Functional enrichment is performed using ToppGene, enabling pathway, disease, and drug-target annotation of gene sets. Literature reasoning is supported through the PubTator3 and EuropePMC APIs, allowing entity-aware retrieval across more than 11 million full-text PubMed Central articles and over 47 million PubMed abstracts and bioRxiv and medRxiv preprints. Clinical trial information is retrieved via the ClinicalTrials.gov API. For all external queries, input terms undergo ontology-based expansion and synonym resolution prior to submission, improving recall while preserving biological specificity. Drug Connectivity analysis is performed through iLINCS, enabling connectivity mapping between disease-associated transcriptional signatures and chemical or genetic perturbation profiles.

#### 4.3.4 Workflow control, context management, and safeguards

LungChat exposes a predefined set of workflow modes namely *Refine Question*, *Explore Data*, *Review Literature*, *Informed Analysis*, and *Test Hypothesis*. These constrain agent behavior, tool selection, and response synthesis to align with different stages of scientific inquiry. Metadata retrieval is performed in a targeted manner, loading only task-relevant fields into context to prevent overload during multi-step analytical workflows.

Short-term conversational state and intermediate workflow outputs are managed using a Redis-backed state store, enabling robust persistence across long analytical sessions. The store is a Redis Stack instance partitioned into separate logical databases for LangGraph conversation checkpoints, interface state and analytical artifacts, and per-user rate-limiting counters, with append-only persistence so that sessions survive restarts and can be resumed or replayed. To ensure analytical integrity, the supervisor agent applies layered safeguards against prompt injection and adversarial inputs, including prompt-level validation and model-based content filtering for both text and voice inputs. These controls ensure that analytical workflows remain grounded in validated tools and clearly defined scientific objectives.

### 4.4 Multi-agent architecture

#### 4.4.1 Agent roles and responsibilities

LungChat employs five specialized agents, each designed for a distinct aspect of multi-omics analysis (Table 2). The **Supervisor Agent** serves as the central orchestrator, determining optimal delegation strategies and possessing unique capabilities including dynamic analytical code execution and direct access to aggregated expression data. The **Single-Cell Agent** specializes in cell-level transcriptomic analysis, operating on pre-processed datasets with computed statistics for rapid interactive exploration. The **Spatial Agent** handles tissue-level analyses including spatial gene expression mapping and cell-cell communication inference across multiple platforms. The **Research Agent** bridges internal data with external knowledge through literature search, functional enrichment analysis, and drug-gene connectivity mapping. The **Atlas Agent** provides structured LungMAP/ontology access to domain-specific resources and controlled vocabularies.

#### 4.4.2 Inter-agent communication and execution model

Agent coordination follows a stateless delegation model designed for reproducibility and modularity. Sub-agents execute independently without access to conversation history or other agents’ intermediate reasoning; they receive only the delegated task specification and return self-contained responses. This design limits the influence of unrelated conversation history and improves the consistency and reproducibility of subagent execution.

The supervisor implements parallel task execution for independent analytical operations. When a query decomposes into non-dependent sub-tasks, these execute concurrently, significantly reducing response latency. Dependent tasks execute sequentially with results from prior steps informing subsequent operations. Task execution follows a managed lifecycle with explicit states: *pending*, *in_progress*, and *completed*. Failed tasks trigger supervisor-mediated retry with parameter adjustment before graceful degradation with informative error reporting.

To support inter-agent collaboration and long-horizon workflows, LungChat employs a shared virtual filesystem serving as a common workspace for intermediate results and analysis outputs. When tool executions produce outputs exceeding a configurable size threshold, results are automatically materialized as filesystem outputs and replaced in context with lightweight file references. All agents have read and write access to the shared workspace, enabling downstream agents to inspect, extend, or refine outputs produced by upstream analyses. The workspace is thread-scoped: each session writes to an isolated output namespace, and file access is mediated by a filesystem backend that resolves artifact references, blocks path traversal and symbolic-link escapes, and enforces size limits, preserving per-user and per-thread isolation across concurrent sessions.

The stateless delegation model serves a dual purpose beyond modularity: it implements context engineering principles that mitigate known limitations of long-context language model reasoning [15]. Each specialist agent operates in a scoped context window containing only its domain-relevant tools and instructions, preventing the cross-domain parameter leakage and instruction dilution that arise when a single agent must reason over all 51 tools simultaneously. Sub-agents are invoked without conversation history, ensuring that reasoning quality at turn n of a session is structurally independent of prior turns, a property we term *stale-context resistance*. This design aligns with recent findings that multi-agent collaboration through scoped worker contexts outperforms monolithic single-agent approaches on long-context tasks [30] and recommended practices for managing context degradation in agentic systems [2].

### 4.5 Tool-augmented reasoning

#### 4.5.1 Tool abstraction layer

LungChat augments language model reasoning with a structured library of analytical tools organized into four functional operator classes: **Analysis Operators** perform statistical and computational procedures including ToppGene for functional enrichment testing and iLINCS for drug-gene perturbation connectivity analysis, alongside differential expression and pathway overlap operators; **Visualization Operators** generate publication-ready figures in both raster and vector formats; **Knowledge Operators** interface with external resources including biomedical literature databases, clinical trial registries, and gene annotation services; **Data Access Operators** provide structured access to consortium data APIs and curated omics datasets.

#### 4.5.2 Hybrid tool selection mechanism

With 51 tools across 4 operator classes, exhaustive evaluation of all tools for each query is computationally prohibitive and degrades response quality through irrelevant context. We implement a two-stage hybrid selection mechanism balancing broad recall with precise final selection.

Given a query q and tool library 𝒯, the selection process is:

(1) $${𝒯}_{\text{selected}}=LLM–Refine\left(Top–K\left({sim}_{vec}(q,𝒯)+\lambda \cdot BM25(q,𝒯)\right),k_{max}\right)$$

where ${sim}_{\text{vec}}$ computes semantic embedding similarity and BM25 provides lexical keyword matching. In practice, λ=0.3 balances semantic and lexical signals, and K=15 defines the initial candidate pool. The candidate pool is then refined by a lightweight language model to select the final $k_{\text{max}}$ tools (typically 3) based on query-tool relevance reasoning. This hybrid approach captures both semantic similarity for paraphrased queries and lexical overlap for specific technical terms, substantially reducing token consumption while maintaining selection accuracy.

### 4.6 Multi-omics data integration

#### 4.6.1 Data modalities

LungChat integrates three complementary omics modalities through a unified query interface, as summarized in Table 1. **Single-cell RNA-seq** data comprises 39 datasets totaling 2.8 million cells spanning fetal development, healthy adult tissue, and 15 disease contexts including idiopathic pulmonary fibrosis, COPD, and COVID-19, with pre-computed differential expression statistics and harmonized cell type annotations across common expression schemas. **Spatial transcriptomics** data includes IPF dataset across both Visium-HD and Xenium platform types capturing spatially-resolved gene expression with pre-computed cell-cell communication objects. **Pseudobulk expression** data provides aggregated views across cell types and conditions in a queryable warehouse with comprehensive differential expression signatures supporting cross-dataset comparisons.

#### 4.6.2 Ontology-grounded metadata harmonization

Metadata harmonization is performed by mapping heterogeneous author-provided annotations to standardized biomedical ontologies including CL, UBERON, MONDO, EFO, and HANCESTRO. This approach achieves robust resolution of heterogeneous annotations to canonical ontology terms, enabling consistent cross-dataset queries. All ontology mappings are versioned and documented to ensure consistent interpretation. Author-provided labels are resolved to canonical ontology identifiers and stored alongside the harmonized expression warehouse in a SQLite catalog with full-text (FTS5) indices over cell-type, disease, assay, developmental-stage, sex, and ancestry vocabularies, enabling fuzzy and synonym-aware term matching. At query time, metadata fields are retrieved on demand and search terms are expanded across ontology ancestors and descendants before warehouse or external-API submission, so that a single disease or cell-type term transparently captures its subtypes and related annotations across heterogeneously labeled datasets.

### 4.7 Direction-Aware Repurposing and Targeting (DART)

To enable cell-type-resolved therapeutic prioritization, we introduce DART, a method that distinguishes perturbations reversing disease-associated transcriptional programs from those that reinforce them. Unlike conventional connectivity analyses producing compound-level scores aggregated across heterogeneous cell populations, DART preserves cell-type resolution throughout analysis, allowing antagonistic effects across lineages to be explicitly identified.

#### Directional scoring.

Given a disease differential expression signature $\Delta_{d}(c)=\{\left(g_{i},{log}_{2}\;{FC}_{i}^{d}\right)\}$ for cell type c and a perturbation-induced transcriptional signature $\Delta_{p}=\{\left(g_{j},{log}_{2}\;{FC}_{j}^{p}\right)\}$, DART converts fold changes to binary directional indicators (+1 for upregulation, −1 for downregulation). For overlapping genes $G_{\text{overlap}}=G_{d}\cap G_{p}$, genes are classified by directional concordance:

(2) $$G_{\text{reversed}}=\{g\in G_{\text{overlap}}|sign({log}_{2}\;{FC}_{g}^{d})\times sign({log}_{2}\;{FC}_{g}^{p})<0\}$$

(3) $$G_{\text{amplified}}=\{g\in G_{\text{overlap}}|sign({log}_{2}\;{FC}_{g}^{d})\times sign({log}_{2}\;{FC}_{g}^{p})>0\}$$

DART computes two heuristic metrics: Pearson correlation on binary directions $\rho (c)={corr}_{\text{Pearson}}\left({dir}_{d},{dir}_{p}\right)$ and reversal rate $R(c)=\left|G_{\text{reversed}}\right|/\left|G_{\text{overlap}}\right|$, where negative ρ and high R indicate therapeutic reversal. Perturbations are ranked by reversal rate (reversal mode) or its complement (amplification mode), enabling prioritization of compounds that oppose or reinforce disease programs. Directional stratification by disease-upregulated versus disease-downregulated genes enables cell-type-specific safety profiling. DART is implemented as a composable analysis operator operating on precomputed signatures and can be applied wherever matched disease and perturbation transcriptional profiles are available.

**Algorithm 1:** Query Decomposition and Execution

**Require:** Query q, Agent set 𝒜, Tool library 𝒯	
**Ensure:** Response ℛ=(𝒱,ℐ,𝒜)	
1:	plan ← Decompose(*q*)
2:	**for** task ∈ plan.parallel_tasks **do**
3:	agent ← SelectAgent(task)
4:	Spawn(task, agent)
5:	**end for**
6:	**for** task ∈ plan.sequential_tasks **do**
7:	result ← Execute(task)
8:	plan ← UpdatePlan(plan, result)
9:	**end for**
10:	results ← Await(plan.all_tasks)
11:	ℛ ← Synthesize(results)
12:	**return** ℛ

### 4.8 Iterative scientific workflows

#### 4.8.1 Planning and task decomposition

Complex scientific queries require structured decomposition into executable sub-tasks. LungChat implements explicit planning through a task decomposition mechanism that transforms natural language queries into coordinated analytical workflows, see Algorithm 1.

The Decompose function employs few-shot prompting with examples of query-to-task mappings. SelectAgent uses rule-based routing based on task keywords with LLM fallback for ambiguous cases. The planning mechanism identifies task dependencies to maximize parallel execution. The supervisor can dynamically revise plans based on intermediate results - if initial analysis reveals unexpected patterns, subsequent tasks can be adjusted or additional analyses spawned without requiring user intervention.

#### 4.8.2 Planning tool

Complex biomedical workflows often require significant computational time, creating a “black box” latency period that erodes user trust. To address this, LungChat implements a real-time task synchronization layer. When executing multi-step protocols, the Supervisor agent actively maintains a structured state object (todos) that maps the analysis plan to visible UI elements. This state is synchronized via a low-latency Redis layer to the frontend, transforming the waiting period into an interactive progress log. Users see the agent decompose their query into discrete biological tasks (e.g., “1. Query Omics Warehouse”, “2. Perform Enrichment”), mark them as in_progress, and resolve them to completed in real-time. This mechanism provides immediate visual verification that the agent is executing the intended logic, distinguishing productive “reasoning time” from system stalls.

#### 4.8.3 Provenance-tracked reproducibility and memory

Scientific analysis requires reproducibility beyond context. LungChat structures all outputs as persistent outputs with complete provenance: **Configuration Outputs** capture all input parameters, data filters, and analysis settings as JSON; **Visualization Outputs** include figures in multiple formats (PNG, PDF) with accompanying data tables (TSV); **Execution Traces** document the sequence of analytical operations, agent delegations, and intermediate results; **Session Reports** provide automated summaries of analytical workflows and key findings, see Appendix C for an example. In addition, each analytical step emits a UID (code_used_*.py or code_used_*.R) containing the exact implementation source and the resolved parameters used for that run, so that any figure or table can be regenerated independently of the conversational interface. Code, configurations, and input files can be downloaded and re-run by the user locally to reproduce results or the JSON directly supplied back to LungChat to replicate or modify prior analyses.

Beyond single-session analysis, LungChat maintains user-specific long-term analytical context across sessions, storing structured research state including previously analyzed genes, selected cell types, and references to generated outputs. Memory retrieval is scoped to analytical context and does not influence model parameters or tool execution logic, ensuring reproducibility while enabling cumulative scientific workflows. Extended analyses exceeding context capacity employ dynamic compression using a dedicated summarization module, preserving recent interactions while compressing prior context. Combined with filesystem-based eviction of large tool outputs, this strategy decouples analytical data volume from context length.

### 4.9 Implementation and inference regime

LungChat operates entirely at inference time using frozen foundation models. No task-specific training, fine-tuning, or gradient updates are performed on any model components. All capabilities including multi-agent coordination, tool selection, and response synthesis emerge from prompt engineering, structured tool augmentation, and orchestration logic. The system employs Claude Sonnet 4.6 and GPT-4 class models with long-context windows (≥128,000 tokens). Tool selection uses low-temperature decoding (set 0.1), while response synthesis uses moderate temperature (set 0.7). This design enables rapid capability updates through prompt and tool modifications without retraining, while leveraging the broad knowledge encoded in pre-trained foundation models.

## Supplementary Material

Supplement 1

## Figures and Tables

**Figure 1:**
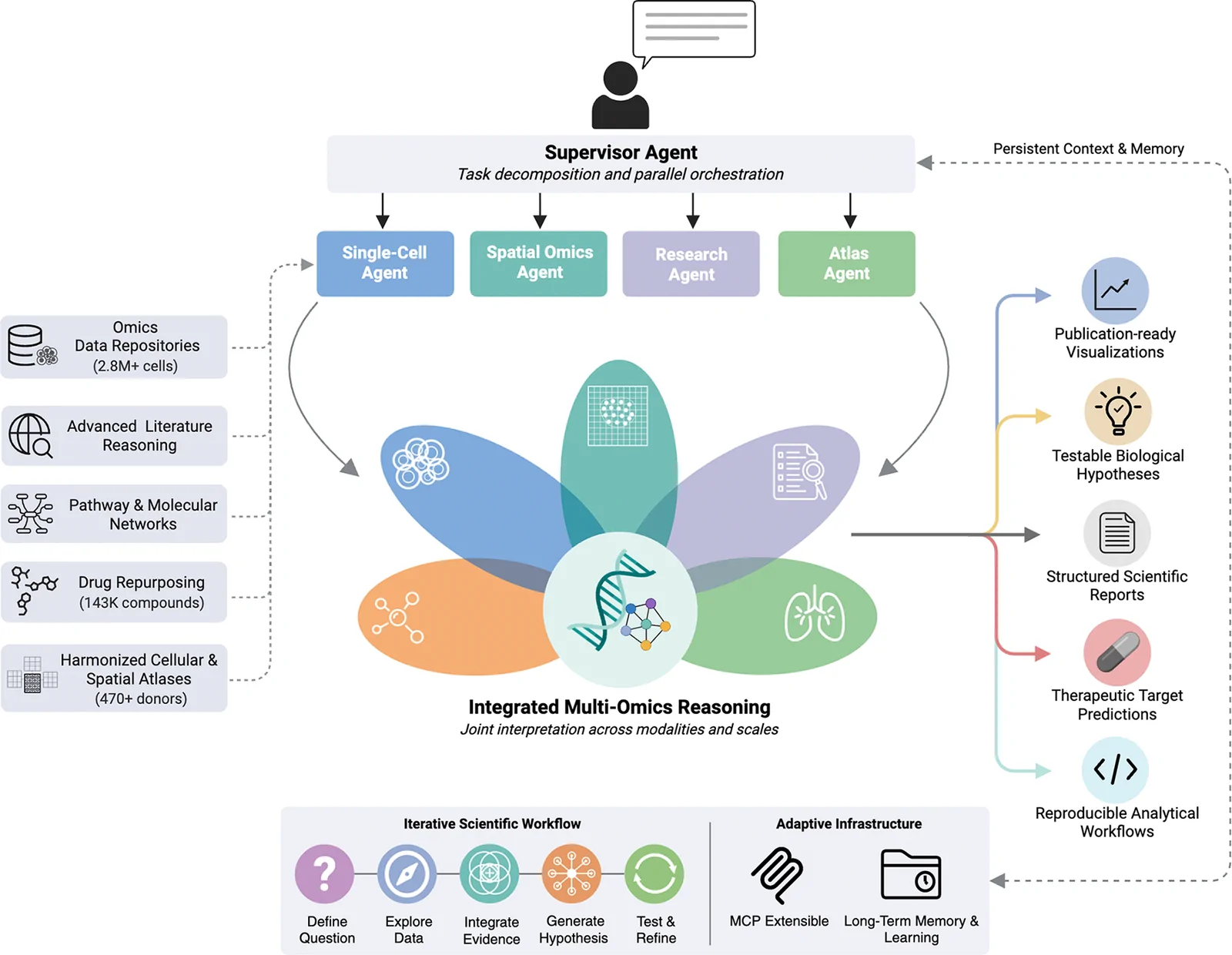
LungChat system architecture and workflow. A hierarchical multi-agent system integrating conversational AI with multi-omics analysis. The Supervisor Agent orchestrates four specialized subagents (Single-Cell, Spatial, Research, Atlas) that access curated data repositories (scRNA-Seq, spatial transcriptomics, drug compounds, regulatory networks, literature) to generate publication-ready outputs including visualizations, testable hypotheses, therapeutic predictions, and reproducible workflows. The system supports iterative scientific workflows (Define → Explore → Integrate → Generate → Test & Refine) with long-term memory and MCP-extensible infrastructure for adaptive tool integration.

**Figure 2:**
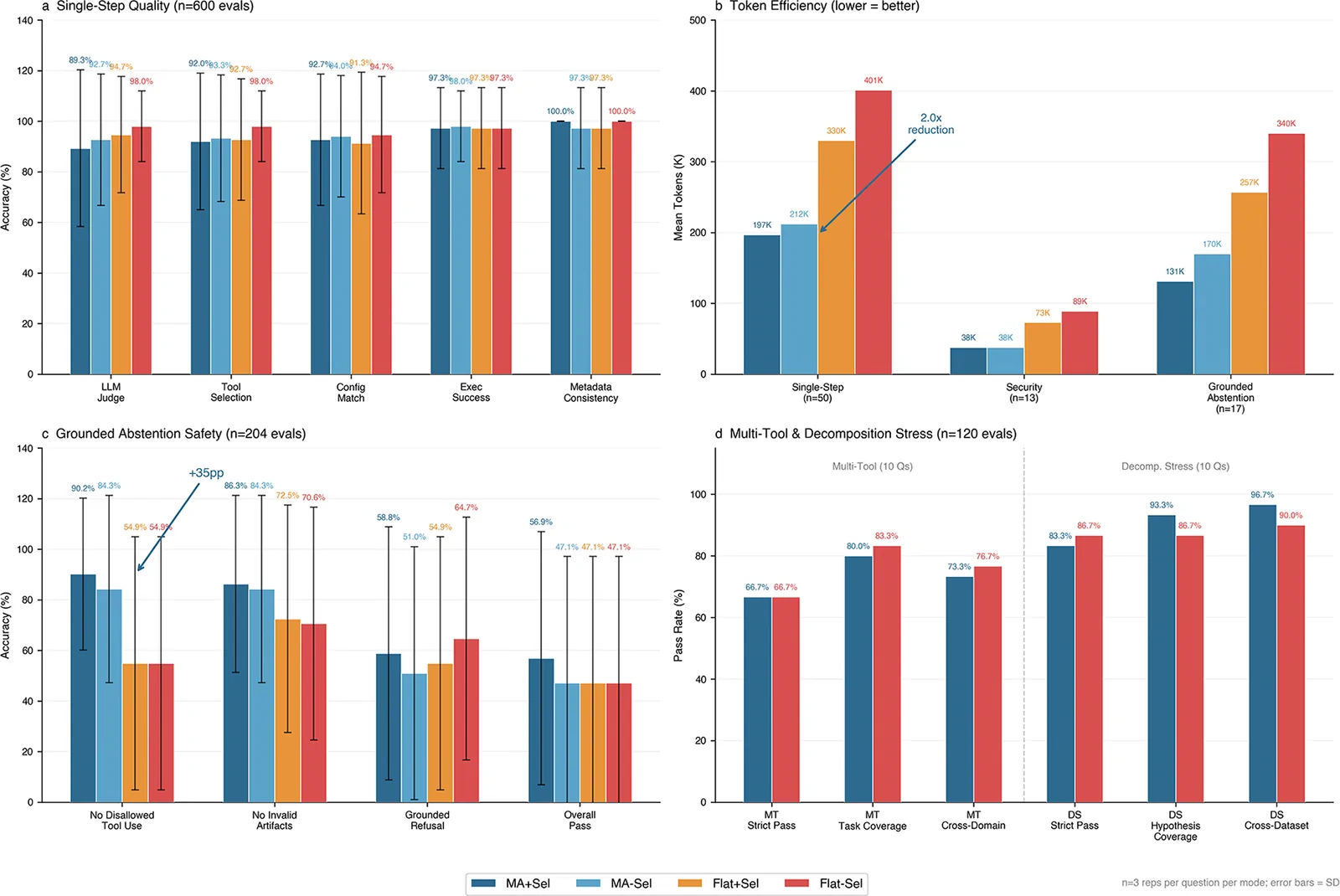
Architecture ablation evaluation across all five tracks (1,080 evaluations). (a) Single-step quality across four ablation configurations (50 queries × 3 reps × 4 modes = 600 evaluations), (b) mean token consumption per query across three evaluation tracks (lower is better), (c) grounded abstention safety metrics (17 queries × 3 reps × 4 modes = 204 evaluations), showing the +35pp improvement in disallowed-tool blocking for the multi-agent configuration, and (d) multi-tool orchestration and decomposition-stress results (20 queries × 3 reps × 2 modes = 120 evaluations), demonstrating architecture-neutral quality on complex tasks.

**Figure 3:**
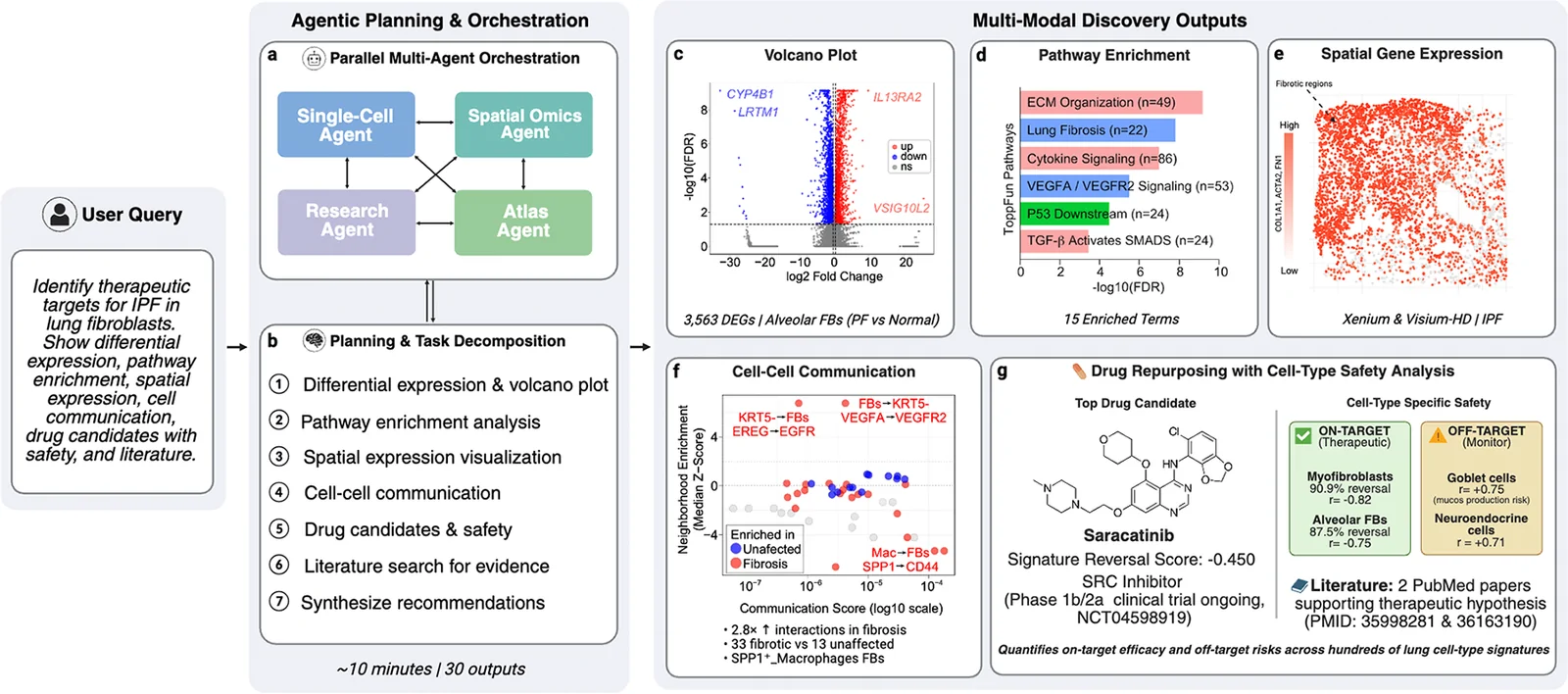
Automated therapeutic target and drug prioritization for pulmonary fibrosis. (a) LungChat parallel multi-agent orchestration with supervisor-coordinated task delegation. (b) Planning and task decomposition for reproducible workflows. (c) Ranking of up- and down-regulated genes in pulmonary fibrosis alveolar fibroblasts. (d) Gene-set enrichment (ToppGene) identifying known druggable pathways in pulmonary fibrosis. (e) Spatial transcriptomic localization of fibrotic markers. (f) Prediction of receptor-ligand interactions ranked by cell-type spatial proximity in pulmonary fibrosis versus controls (Xenium) cell-cell communication strength. (g) Drug repurposing with DART cell-type safety analysis: Saracatinib identified as top candidate. Off-target monitoring identified goblet cells and neuroendocrine cells.

**Figure 4:**
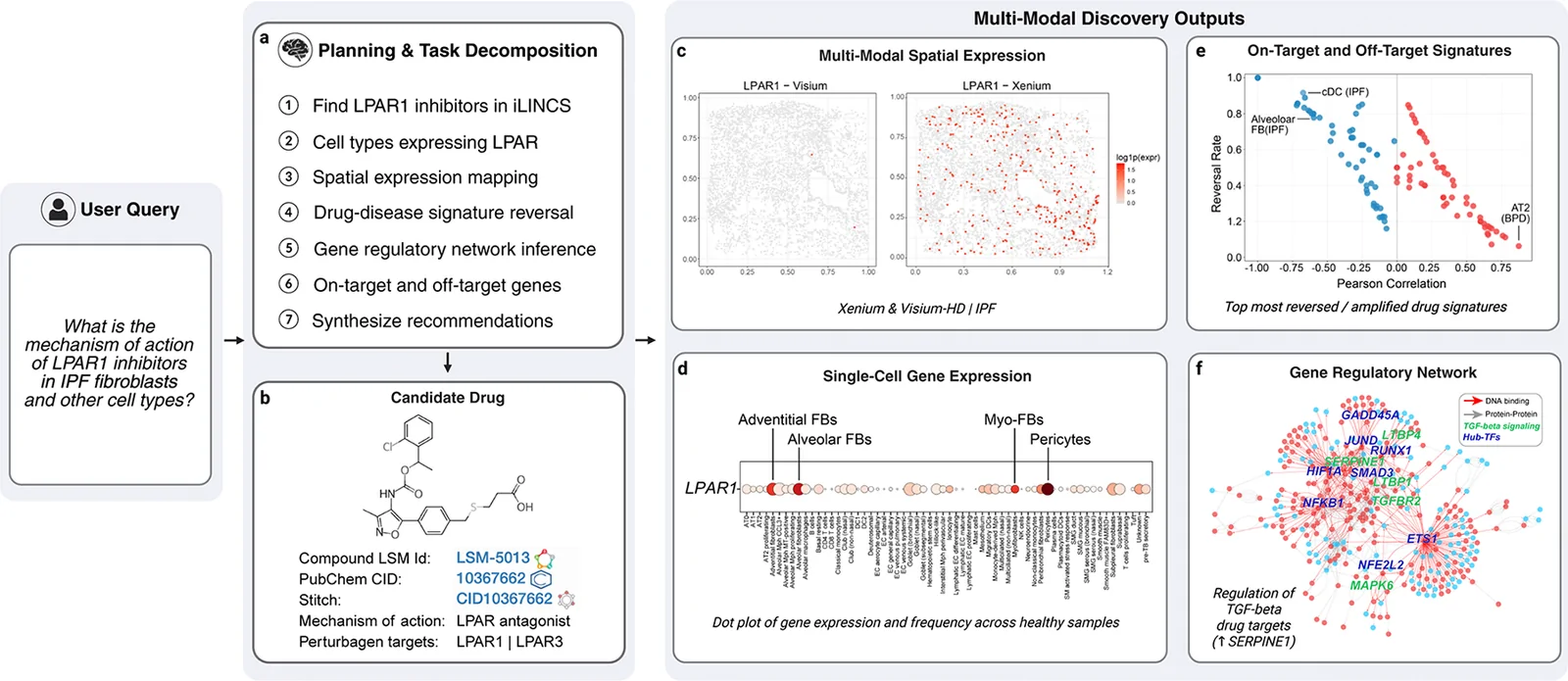
Resolving the regulatory mechanisms and cell targets of new therapies. (a) Planning and task decomposition to define diverse mechanisms of action for LPAR1 inhibitors in pulmonary fibrosis. (b) Identification of LPAR1 inhibitors in iLINCS with in vitro gene expression responses. (c) Comparison of the spatial distribution LPAR1 in Xenium and Visium-HD (same slide - Spatial Agent). (d) Expression and frequency of LPAR1 across adult healthy lung cell populations (HLCA - Single Cell Agent). (e) DART prediction of the top impacted lung disease, technology and developmental perturbations (Omics Data Repository), reversed or further amplified by in vitro LPAR agonist treatment (iLINCS). (f) Inferred gene regulatory network from annotated transcription factor (TF) target and pathway interactions (NetPerspective), with highlighted major TF nodes and annotated TGF-beta signaling responsive genes (ToppGene).

**Table 1:** LungChat data and system coverage.

Resource	Coverage

Single-cell datasets	39 (2.8M cells)
Spatial datasets	1 (Visium-HD, Xenium)
Disease contexts	15
Specialized tools	51
Precomputed DEG signatures	579

**Table 2:** Agent specifications.

Agent	Role	Analytical Scope

Supervisor	Query routing, result synthesis, code execution	Cross-modal coordination
Single-Cell	Differential expression, cell characterization	scRNA-seq transcriptomics
Spatial	Tissue organization, cell communication	Spatial transcriptomics
Research	Literature integration, enrichment, connectivity	External knowledge
Atlas	Ontology queries, vocabulary lookup	Domain databases

## Data Availability

This study analyzes previously generated single-cell and spatial transcriptomics datasets available through public or consortium repositories. The Human Lung Cell Atlas v2 dataset is available through CELLxGENE under accession 6f6d381a-7701–4781-935c-db10d30de293. The fetal lung cell atlas is available through CELLxGENE under accession 2d2e2acd-dade-489f-a2da-6c11aa654028. Additional LungMAP consortium datasets are available under accessions LMEX0000004400 and LMEX0000004401. Spatial transcriptomics data are available from the Gene Expression Omnibus under accession GSE276945, including GSM8505452. Derived analysis outputs, configuration files, benchmark summaries, and figure source data generated for this study are provided in the manuscript appendices and supplementary materials, or are available upon reasonable request subject to applicable repository, consortium, and institutional restrictions.

## Code Availability

The LungChat interface described in this manuscript is deployed at https://chat.lungmap.net. The complete production application codebase, including deployment-specific frontend, infrastructure, authentication, and operational components, is not publicly released at this time. Analysis code, configuration examples, DART output tables, benchmark summaries, and other reproducibility materials sufficient to support the analyses reported in this manuscript are available upon reasonable request, subject to institutional approval.
