# Supplementary material for: A Conversational Multi-Agent AI System for Integrated Multi-Omics Analysis and Biomedical Discovery
Source: bioRxiv. 2026 Aug 14:2026.08.08.743577. Preprint. [Version 1] doi: 10.64898/2026.08.08.743577 (PMC13640590; doi:10.64898/2026.08.08.743577)
Supplement: Supplementary file 1 [file NIHPP2026.08.08.743577v1-supplement-1.pdf]

bioRxiv preprint doi: https://doi.org/10.64898/2026.08.08.743577; this version posted August 14, 2026. The copyright holder for this preprint (which was not certified by peer review) is the author/funder, who has granted bioRxiv a license to display the preprint in perpetuity. It is made available under a CC-BY 4.0 International license.

A Dataset curation and preprocessing

A.1 Single-cell RNA-seq

Four independent single-cell RNA-Seq collections were integrated into LungChat's corpus of initial available datasets. The largest of these dataset collections consist of 2.2 million cells from 486 lungs from Human Lung Cell Atlas (HLCA) version 2 [25] object in CellxGene (accession: 6f6d381a-7701-4781-935c-db10d30de293 https://cellxgene.cziscience.com/collections/6f6d381a-7701-4781-935c-db10d30de293). HLCA is a collection of reprocessed raw droplet sequencing data 35 single-cell studies, spanning 14 dis-tinct disease entities. To efficiently assess and analyze this data, the R library SuperCell v.1.0 was used to produce 50,520 metacells from the HLCA, separately for each individual donor and HLCA consortia annotated cluster (ann_finest_level). A lung cell atlas of fetal development was also obtained from CellxGene (accession: 2d2e2acd-dade-489f-a2da-6c11aa654028 https://cellxgene.cziscience.com/collections/2d2e2acd-dade-489f-a2da-6c11aa654028), spanning 5-22 post-conception weeks [8]. Two additional LungMAP consortia datasets were provided to LungChat, comprising infant lungs at the time of death from bronchopulmonary dysplasia or other causes (controls) (LungMAP.net accession: LMEX0000004400 https://www.lungmap.net/dataset/?dataset_id=LMEX0000004400, LMEX0000004401 https://www.lungmap.net/dataset/?dataset_id=LMEX0000004401) [24]. Cell annotations for each dataset include author curated at different levels of resolution and Cell Ontology unique IDs. All datasets are formatted by LungChat pre-processing as anndata H5AD objects, from source H5AD or RDS, using developed automated processing workflows (GitHub). This workflow retains author provided UMAP coordinates (averaged for metacells) and harmonized sample metadata (CellxGene). The preprocessing workflow further produces normalized expression using the Scanpy Python package (natural log of counts per 10,000

bioRxiv preprint doi: https://doi.org/10.64898/2026.08.08.743577; this version posted August 14, 2026. The copyright holder for this preprint (which was not certified by peer review) is the author/funder, who has granted bioRxiv a license to display the preprint in perpetuity. It is made available under a CC-BY 4.0 International license.

reads), translates Ensembl primary identifiers to gene symbols (Ensembl 100), computes cell population marker gene and covariate differential gene expression versus appropriate control conditions (wilcoxon, FDR corrected) and retains DEGs with fold > 1.2 and Mann-Whitney U test $p < 0.05$ (FDR adjusted) in uns["rank_genes_groups"]. Study-specific gene signatures are stored for later comparison in LungChat. Each anndata object is augmented with a database of protein-protein and protein-DNA interactions from the AltAnalyze NetPerspective database [28], for fast retrieval and display of inferred gene regulatory networks in LungChat.

A.2 Spatial transcriptomics

Image-based (10x Genomics Xenium) and whole transcriptome (10x Genomics Visium-HD) spatial transcriptomics from a collection of human healthy and IPF lungs were obtained from the Gene Expression Omnibus database (GSE276945 https://www.ncbi.nlm.nih.gov/geo/query/acc.cgi?acc=GSE276945). Each patient sample corresponds to a region of interest (ROI), tiled on the Xenium (343 gene probes) or Visium slide (18,085 genes, GSM8505452 https://www.ncbi.nlm.nih.gov/geo/query/acc.cgi?acc=GSM8505452). For Xenium samples (n=35), author generated cell segmentation (1.6 million cells), curated cell annotation (Final_CT) and niche annotation (TNiche and CNiche) were retained, along with sample level metadata (i.e., sex, age). Segmented cell annotations were augmented with Robust Cell Type Decomposition (RCTD) from the spacexr package that was applied to derive supervised cell annotations for RCTD-classified singlets using the human CellRef transcriptome as a reference. For Visium-HD, as no author annotation for 8μm spots were provided, clusters, the same RCTD protocol was applied for Visium spots, to generate annotations.

To derive hybrid multi-modal cell annotations from the integration of matching Xenium and Visium samples, we reoriented, scaled and aligned Xenium segmented map (query) to Visium (reference) coordinates, considering unique cell/spot correspondences (dplyr package in R). For downstream integrated analyses, all cell/spots were downsampled to those with 1:1 correspondences using the find nearest neighbor function (FNN) package and get.knnx() function. Mapping was verified using COL1A1 expression, which was highly expressed by both platforms. Cluster annotations were mapped across 1:1 mapped cells/spots across platforms to define the most stable cell populations using a new spatial library within the scTriangulate package (https://github.com/frankligy/scTriangulate). scTriangulate [14] uses a game-theory approach to compare distinct stability metrics (marker gene expression, reclassification) for each single-cell cluster, separately for each modality evaluated. In this instance, all Xenium and Visium cell or niche clusters were evaluated using the integrated object, with RNA values and spatial coordinates from each platform considered as separate modalities. A final set of 29 multimodal stable clusters were defined from this analysis and stored in the integrated RDS object for LungChat analysis.

To infer cell-cell communication networks, we applied the python package Squidpy to assess cell-type spatial proximity via neighborhood enrichment (z-score) and CellChat to infer cell-cell and receptor-ligand interactions. These analyses were pre-computed using the Xenium dataset considering control (less affected) and IPF (more affected) samples. Both Squidpy and CellChat were applied to different annotation layers, namely author (Final_CT), cellular niches (CNiche, TNiche) and Final_CT annotations that subdivide individual niches. Computed results were stored in Seurat RData objects for direct access by LungChat.

B Tool reference and output specifications

B.1 Tool scope

This appendix provides a capability-level reference of analytical, external API, and knowledge tools used by the Supervisor, Single-Cell, Spatial, and Research agents (Atlas Agent excluded).

bioRxiv preprint doi: https://doi.org/10.64898/2026.08.08.743577; this version posted August 14, 2026. The copyright holder for this preprint (which was not certified by peer review) is the author/funder, who has granted bioRxiv a license to display the preprint in perpetuity. It is made available under a CC-BY 4.0 International license.

B.2 Capability-level tool catalog

Table B1: Capability-level reference of analytical, external API, and knowledge tools used by the Supervisor, Single-Cell, Spatial, and Research agents (Atlas Agent excluded), including output formats and descriptions.

Tool	Output format	Description
**Supervisor Agent Tools**		
`query_pseudobulk_expression_data_tool`	JSON	Query pseudobulk omics warehouse (DEG lookup, aggregation, comparisons, signature overlap, drug–cell-type impact)
`execute_code_tool`	JSON	Run Python or R in sandbox
**Single-Cell (scRNA-seq) Agent Tools**		
`plot_heatmap`	JSON, PDF, PNG, TSV	Clustered heatmap of gene expression across cell types/conditions.
`plot_radar`	JSON, PDF, PNG	Radar/spider chart of cell type composition across conditions.
`plot_cell_frequency`	JSON, PDF, PNG	Box/violin of cell frequencies per donor; Mann-Whitney U.
`plot_volcano`	JSON, PDF, PNG	Volcano: log2FC vs FDR; top genes labeled.
`plot_dotplot`	JSON, PDF, PNG	Dot plot: % expressing (size), mean expression (color) across cell types.
`plot_violin`	JSON, PDF, PNG	Violin of one gene expression across cell types/conditions.
`plot_venn`	JSON, PDF, PNG	Venn diagram of gene set overlaps (2–3 sets).
`plot_upset_genes`	JSON, PDF, PNG	UpSet plot for 4+ gene set overlaps.
`plot_umap`	JSON, PDF, PNG	UMAP 2D embedding; optional gene overlay.
`plot_network`	JSON, PDF, PNG	Gene–gene interaction/regulatory network.
`plot_gene_correlation`	JSON, PDF, PNG, TSV	Pearson correlation of genes with query gene.
`generate_stats`	JSON, TSV	Export DEG/marker statistics.
**Spatial Agent Tools**		
`plot_spatial`	JSON, PDF, PNG	Spatial plot of cell annotations (Final_CT, TNiche, CNiche, etc.) on coordinates.
`plot_visualize_celltypes_spatial_distribution`	JSON, PDF, PNG	Multi-panel spatial distribution of cell types across samples/groups.
`plot_compare_spatial_rna_with_histology_quadpanel`	JSON, PDF, PNG	4-panel: Visium-HD, Xenium, H&E, DAPI.

bioRxiv preprint doi: https://doi.org/10.64898/2026.08.08.743577; this version posted August 14, 2026. The copyright holder for this preprint (which was not certified by peer review) is the author/funder, who has granted bioRxiv a license to display the preprint in perpetuity. It is made available under a CC-BY 4.0 International license.

Tool	Output format	Description
`plot_umap`	JSON, PDF, PNG	UMAP colored by annotation (spatial data).
`plot_visualize_gene_expression_spatial`	JSON, PDF, PNG	Gene expression on spatial coordinates (Xenium/Visium).
`plot_barchart_top_correlated_visium_xenium_rna`	JSON, PDF, PNG	Bar chart of top correlated genes across Visium/Xenium.
`plot_evaluate_cluster_stability_report`	JSON, PDF, PNG	Report on annotation stability across sources.
`rank_celltype_marker_genes_by_specificity`	JSON, TSV	Rank cell types by marker specificity (t-test/FDR).
`plot_celltype_marker_volcano`	JSON, PDF, PNG	Volcano of marker genes for one cell type.
`plot_compare_markers_across_platforms_dotplot`	JSON, PDF, PNG	Dot plot comparing markers across platforms.
`plot_evaluate_annotation_concordance_dotplot`	JSON, PDF, PNG	Dot plot of annotation concordance.
`plot_scatter_matched_xenium_visium_spots`	JSON, PDF, PNG	Scatter of matched Xenium–Visium spot expression.
`plot_differential_expression_volcano`	JSON, PDF, PNG	Volcano for spatial DEGs between groups.
`plot_cellchat_chord_diagram`	JSON, PDF, PNG	CellChat chord diagram of signaling.
`plot_cellchat_heatmap`	JSON, PDF, PNG	CellChat signaling strength heatmap.
`plot_cellchat_signaling_pathway_network`	JSON, PDF, PNG	CellChat pathway network.
`extract_cellchat_interactions_subset`	JSON, TSV	Extract subset of CellChat interactions.
`plot_squidpy_neighbour_enrichment_heatmap`	JSON, PDF, PNG	Squidpy neighborhood enrichment heatmap.
`plot_squidpy_celltype_neighborhood_enrichment`	JSON, PDF, PNG	Squidpy cell type neighborhood enrichment.
`plot_identify_significant_proportion_changes`	JSON, PDF, PNG, TSV	Proportion changes between groups.
`plot_compare_different_cell_annotations_heatmap`	JSON, PDF, PNG	Heatmap comparing two annotation columns.
`plot_identify_celltypes_enriched_in_niches`	JSON, PDF, PNG	Cell types enriched in niches.
`plot_squidpy_compare_neighborhood_changes`	JSON, PDF, PNG	Compare Squidpy neighborhoods across groups.
`identify_unique_cellchat_interactions_spatial`	JSON, TSV	Unique CellChat interactions per group.
`plot_compare_communication_profiles`	JSON, PDF, PNG	Compare CellChat communication between groups.
**Research Agent Tools**		
`gene_functional_enrichment_tool`	JSON, TSV	ToppGene enrichment (20 categories).

bioRxiv preprint doi: https://doi.org/10.64898/2026.08.08.743577; this version posted August 14, 2026. The copyright holder for this preprint (which was not certified by peer review) is the author/funder, who has granted bioRxiv a license to display the preprint in perpetuity. It is made available under a CC-BY 4.0 International license.

Tool	Output format	Description
`literature_search_tool`	JSON	PubMed + Europe PMC; ranking, preprints, entity extraction. Returns papers, insights.
`clinical_trials_search_tool`	JSON	ClinicalTrials.gov v2; term expansion (MONDO/EFO). Search or retrieve by NCT ID.
`drug_connectivity_tool`	JSON, PDF, PNG, TSV	iLINCS: signature connectivity, compound investigation, gene knockout, similar compounds.
`web_search_tool`	JSON	Google Serper API; fallback for non-biomedical queries. Returns title, snippet, link.
`think_tool`	JSON	Record strategic reflection (progress, gaps, next steps). Returns confirmation.

C Provenance-tracked configuration example


```json
{
  "adata_file": "HLCA_full_superadata_v3_norm_log_deg.h5ad",
  "plot_title": null,
  "cell_type": "Alveolar fibroblasts",
  "disease": "pulmonary fibrosis",
  "top_n": 8,
  "direction": "regulated",
  "cell_type_index": "ann_finest_level",
  "covariate_index": "disease",
  "plot_type": "volcano",
  "thread_id": "d8b70f18-0b56-45fc-a13d-4479e687d2cd",
  "base_url": "http://chat.lungmap.net/lungchat/public/output",
  "tool": "volcano",
  "timestamp": "2026-02-09T07:07:41.925473+00:00",
  "task": "scRNA-seq"
}
```


Figure C1: **Provenance-tracked configuration output for reproducible tool execution.** Example JSON payload automatically saved by LungChat for a differential-expression volcano plot request (dataset, cell type, disease, direction, and tool parameters), enabling deterministic re-execution and auditability of analysis settings as described in Section 4.8.3.

bioRxiv preprint doi: https://doi.org/10.64898/2026.08.08.743577; this version posted August 14, 2026. The copyright holder for this preprint (which was not certified by peer review) is the author/funder, who has granted bioRxiv a license to display the preprint in perpetuity. It is made available under a CC-BY 4.0 International license.

D Capability comparison with related systems

Table D1: Capability-level comparison with related conversational and agentic biomedical AI systems.

Capability	LungChat	Frontier LLMs	CellWhisperer	CompBioAgent / scChat	BioAgents	Biomni
Conversational scientific interface	Strong	Strong	Strong	Strong	Moderate	Strong
Executable omics workflows	Strong	Limited	Limited	Moderate	Moderate	Strong
Multi-agent orchestration	Strong	Limited	Limited	Limited	Strong	Strong
Cross-dataset integration	Strong	Limited	Moderate	Moderate	Moderate	Strong
Single-cell + spatial integration	Strong	Limited	Limited	Limited	Limited	Moderate
Cell-type–resolved drug / perturbation analysis	Strong	Limited	Limited	Limited	Limited	Moderate
Evidence Ranked Literature Mining	Strong	Moderate	Limited	Limited	Moderate	Strong
Provenance-tracked outputs	Strong	Limited	Limited	Limited	Moderate	Moderate
Persistent analytical memory	Strong	Limited	Limited	Limited	Limited	Moderate
Domain-specific depth in lung multi-omics	Strong	Limited	Limited	Limited	Limited	Limited
General-purpose biomedical breadth	Moderate	Strong	Limited	Limited	Moderate	Strong

Direct quantitative benchmarking against competing systems is precluded by non-overlapping analytical capabilities: the end-to-end workflows evaluated here, spanning differential expression, functional enrichment, drug connectivity screening, cell-type–resolved perturbation analysis (DART), and spatial transcriptomics, require integrated tool access that no single competing system currently supports. While several systems offer public deployments (Biomni, CellWhisperer, CompBioAgent), each addresses a subset of these capabilities (Table D1). We therefore provide this qualitative capability comparison alongside a controlled single-agent vs. multi-agent architecture ablation (Section 2.2).

E DART case studies

This appendix provides comprehensive execution provenance for the two DART case studies described in Section 2.3: (1) the IPF Saracatinib discovery, executed as a single-prompt, zero-shot workflow; and (2) the COPD Fluticasone Propionate discovery, executed as a single-prompt, cell-type-resolved zero-shot workflow with per-cell-type iLINCS screening. Both case studies were conducted in fresh sessions with no prior conversation history. All data, tables, and narrative text below are derived directly from the LungChat conversation exports and LangSmith agent execution traces. Figures reference generated outputs archived with the session.

E.1 IPF: Saracatinib Discovery

Supplementary E1. IPF DART Case Study: Single-Prompt Zero-Shot Therapeutic Discovery

User prompt (verbatim).

Identify therapeutic targets and drug repurposing candidates for IPF based on cell-type–specific disease programs in fibroblast populations most associated with fibrosis, comparing diseased and healthy states within those cells, and how these manifest across lung cell types and tissue context, including safety. Prioritize drugs that reverse this program, and for the top candidate, assess its cell-type-specific effects across the lung.

Supervisor decomposition. LungChat's supervisor agent decomposed this single natural-language prompt into six coordinated sub-tasks, executed across three specialized agents with parallel dispatch for independent operations:

1. **Define the disease program:** DEGs in IPF fibroblasts vs. healthy controls (HLCA)

bioRxiv preprint doi: https://doi.org/10.64898/2026.08.08.743577; this version posted August 14, 2026. The copyright holder for this preprint (which was not certified by peer review) is the author/funder, who has granted bioRxiv a license to display the preprint in perpetuity. It is made available under a CC-BY 4.0 International license.

2. **Drug repurposing:** iLINCS screen for compounds reversing the fibroblast signature
3. **Pathway enrichment:** Characterize the fibrotic program biologically (ToppGene)
4. **Cell-type impact:** Map the top drug candidate across all lung cell types (DART)
5. **Spatial context:** Fibroblast niches and ligand-receptor signaling in IPF tissue
6. **Safety synthesis:** Integrate all findings into a prioritized report

Steps 1, 3, and 5 were dispatched in parallel. Steps 2 and 4 were executed sequentially after Step 1 completed.

bioRxiv preprint doi: https://doi.org/10.64898/2026.08.08.743577; this version posted August 14, 2026. The copyright holder for this preprint (which was not certified by peer review) is the author/funder, who has granted bioRxiv a license to display the preprint in perpetuity. It is made available under a CC-BY 4.0 International license.

Complete execution trace.

Step	Agent	Tool(s)	Operation
1	Supervisor	`decompose`	Parses user query into 6 sub-tasks; identifies parallelizable branches
2	Single-Cell	`generate_stats`	Differential expression: Alveolar Fibroblasts, IPF vs. control (HLCA). Produces 3,563 DEGs (FDR < 0.05); 56 up (logFC > 0.5), 55 down (logFC < -0.5) at stringent thresholds
3	Research	`gene_functional_enrichment_tool`	ToppGene enrichment on 56 upregulated and 55 downregulated gene sets. Returns GO Biological Process, Pathway, Disease, and Drug annotations
4	Spatial	`plot_visualize_celltypes_spatial_distribution`, `plot_compare_communication_profiles`	Spatial distribution of fibroblast subtypes (Xenium); CellChat + Squidpy interaction analysis for Activated_Fibrotic_FBs (IPF vs. control). Returns 46 significant interactions, 33 unique to IPF
5	Research	`drug_connectivity_tool`	iLINCS connectivity analysis: 3,563-gene alveolar fibroblast IPF signature screened against 2,383 LINCSCP compounds in reversal mode. Returns ranked drug list
6	Supervisor	`drug_connectivity_tool` (investigate)	Retrieves Saracatinib's own iLINCS compound perturbation signature (976 genes) for downstream DART analysis
7	Supervisor	`query_pseudobulk_expression_data_tool (drug_cell_type_impact)`	Executes DART: correlates Saracatinib signature against 475 lung cell-type/disease signatures across HLCA, BPD_Sun, and HCA_fetal datasets
8	Supervisor	Synthesis	Aggregates all outputs; generates integrated therapeutic report with efficacy, spatial context, and safety profiling

bioRxiv preprint doi: https://doi.org/10.64898/2026.08.08.743577; this version posted August 14, 2026. The copyright holder for this preprint (which was not certified by peer review) is the author/funder, who has granted bioRxiv a license to display the preprint in perpetuity. It is made available under a CC-BY 4.0 International license.

Step 2 output: fibroblast disease program. Analysis of alveolar fibroblasts from the HLCA identified a robust fibrotic gene expression signature comprising **3,563 significant DEGs** (FDR < 0.05). The upregulated program is dominated by extracellular matrix remodeling, cell adhesion, and pro-inflammatory signaling; the downregulated program indicates loss of fibroblast detoxification and quiescence capacity.

Top Upregulated Genes (Active Fibrotic Program):

Gene	Log2FC	Functional Role
IL13RA2	$+9.69$	IL-13 decoy receptor; fibrosis amplifier
THY1	$+6.54$	Fibroblast activation marker (CD90)
NFATC2	$+5.63$	Transcription factor; myofibroblast activation
CPXM1	$+5.59$	Extracellular metalloprotease
IGFBP4	$+5.37$	IGF signaling modulator
SPHK1	$+4.87$	Sphingosine kinase; pro-fibrotic lipid signaling
IL32	$+4.87$	Pro-inflammatory cytokine
SAMD11	$+4.84$	Transcriptional regulator
SERPINE2	$+4.79$	Serine protease inhibitor; ECM remodeling
RUNX1	$+3.79$	Myeloid/fibroblast TF; differentiation driver

Top Downregulated Genes (Lost Homeostatic Program):

Gene	Log2FC	Functional Role
CYP4B1	-32.6	Xenobiotic metabolism; lung-specific detoxification
SAA1	-6.12	Acute phase protein
NKD1	-5.94	Wnt pathway negative regulator
HIF3A	-5.40	Hypoxia response modulator
CCN5	-3.55	Anti-fibrotic matricellular protein
ALDH1A1	-3.14	Retinoid metabolism; fibroblast quiescence
PDGFRA	-1.59	PDGF receptor; fibroblast identity marker

Step 3 output: functional enrichment. ToppGene enrichment of the 56 upregulated genes identified the following top-ranked processes and pathways:

Term	p-value	Genes
Regulation of cell adhesion	2.47×10^{-9}	15/49
Regulation of cell migration	1.36×10^{-8}	16/49
Regulation of cell motility	2.98×10^{-8}	16/49
Positive regulation of cell adhesion	1.06×10^{-7}	—
Regulation of plasminogen activation	1.08×10^{-7}	—
Dissolution of fibrin clot (Reactome)	7.69×10^{-6}	—
Eicosanoid synthesis (WikiPathways)	4.06×10^{-5}	—
HIF-1 transcription factor pathway	4.96×10^{-5}	—
RUNX1-regulated myeloid differentiation	1.94×10^{-4}	—

The downregulated program was enriched for Phase I xenobiotic metabolism (CYP4B1, FMO2, INMT; $p = 6.01 \times 10^{-8}$), amino acid metabolism, and RECK pathway (MMP regulation), indicating loss of normal fibroblast detoxification capacity.

Key druggable targets identified by enrichment: kinases (MAPKAPK2, PIM3, NEK6), receptors (IL1R1, TNFRSF1A, LPAR1, AGTR1), and transcription factors (RUNX1, NFATC1/NFATC2, SOX4, FOXP1).

bioRxiv preprint doi: https://doi.org/10.64898/2026.08.08.743577; this version posted August 14, 2026. The copyright holder for this preprint (which was not certified by peer review) is the author/funder, who has granted bioRxiv a license to display the preprint in perpetuity. It is made available under a CC-BY 4.0 International license.

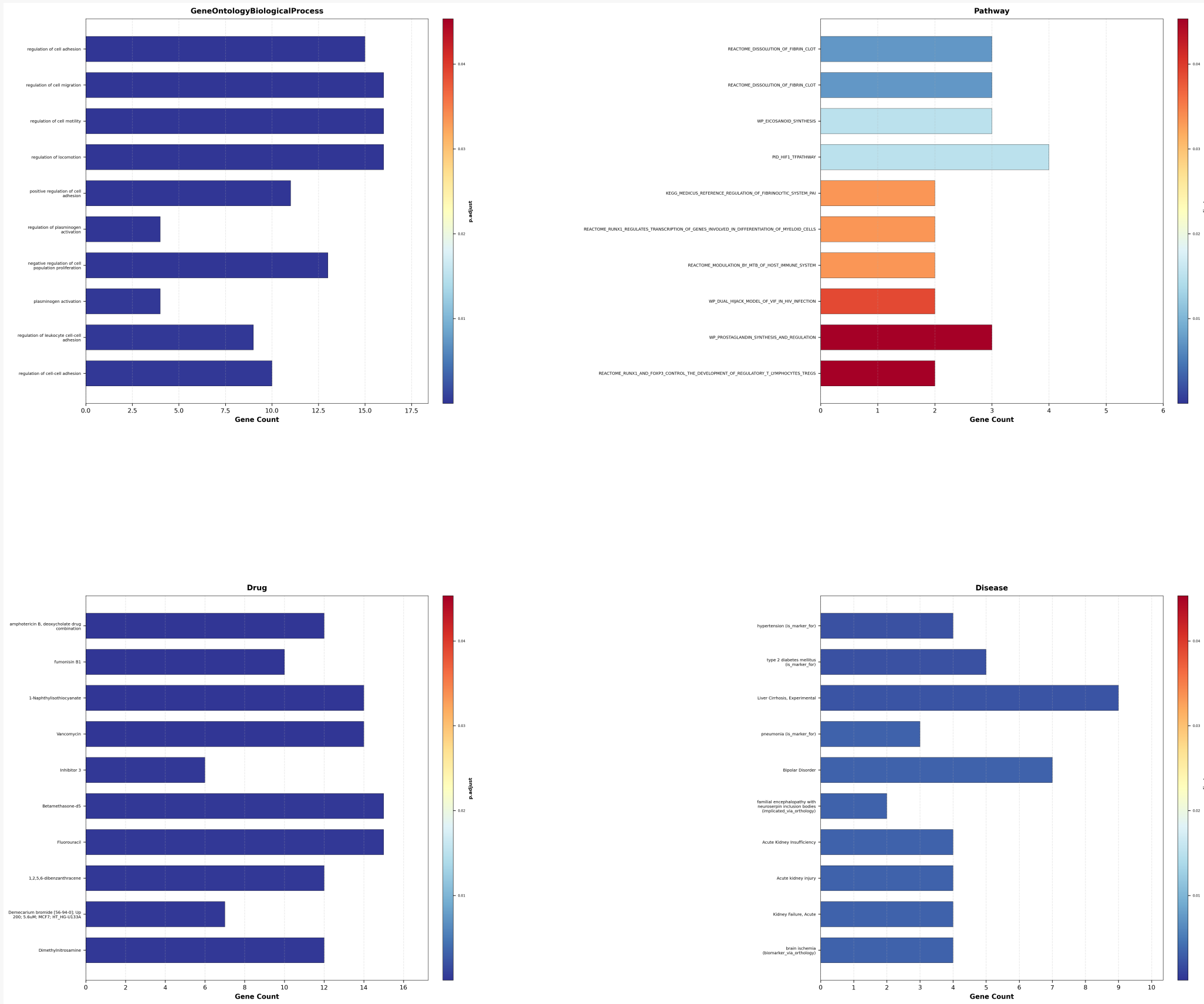

Figure E1: ToppGene functional enrichment of the top 56 upregulated IPF alveolar fibroblast genes. GO Biological Process terms highlight cell adhesion, migration, and HIF-1 signaling; Disease ontology maps to pulmonary fibrosis and related pathologies; Drug associations support kinase inhibitor candidates. All terms FDR-corrected, $q < 0.05$.

Step 4 output: spatial context. Spatial transcriptomics (Xenium, single-cell resolution) revealed seven distinct fibroblast subtypes with segregated niches in unaffected lung tissue: Alveolar_FBs distributed throughout parenchyma, Subpleural_FBs at tissue edges, Myofibroblasts in focal clusters, and Activated_Fibrotic_FBs near remodeling zones. CellChat and Squidpy interaction analysis of Activated_Fibrotic_FBs (IPF vs. control) identified:

- **46 significant interactions** in More_Affected tissue; **33 unique to IPF**
- **Gained in IPF:** Interactions with KRT5^{-}/KRT17^{+} aberrant basaloid cells and capillary populations; monocyte–fibroblast crosstalk amplified
- **Lost in IPF:** Lymphatic and venous endothelial communication; NK cell interactions reduced
- **Top ligand–receptor pairs:** VEGFA–VEGFR2, EREG–EGFR, SPP1–ITGAV/ITGB1, SPP1–CD44, CCL2–ACKR1

bioRxiv preprint doi: https://doi.org/10.64898/2026.08.08.743577; this version posted August 14, 2026. The copyright holder for this preprint (which was not certified by peer review) is the author/funder, who has granted bioRxiv a license to display the preprint in perpetuity. It is made available under a CC-BY 4.0 International license.

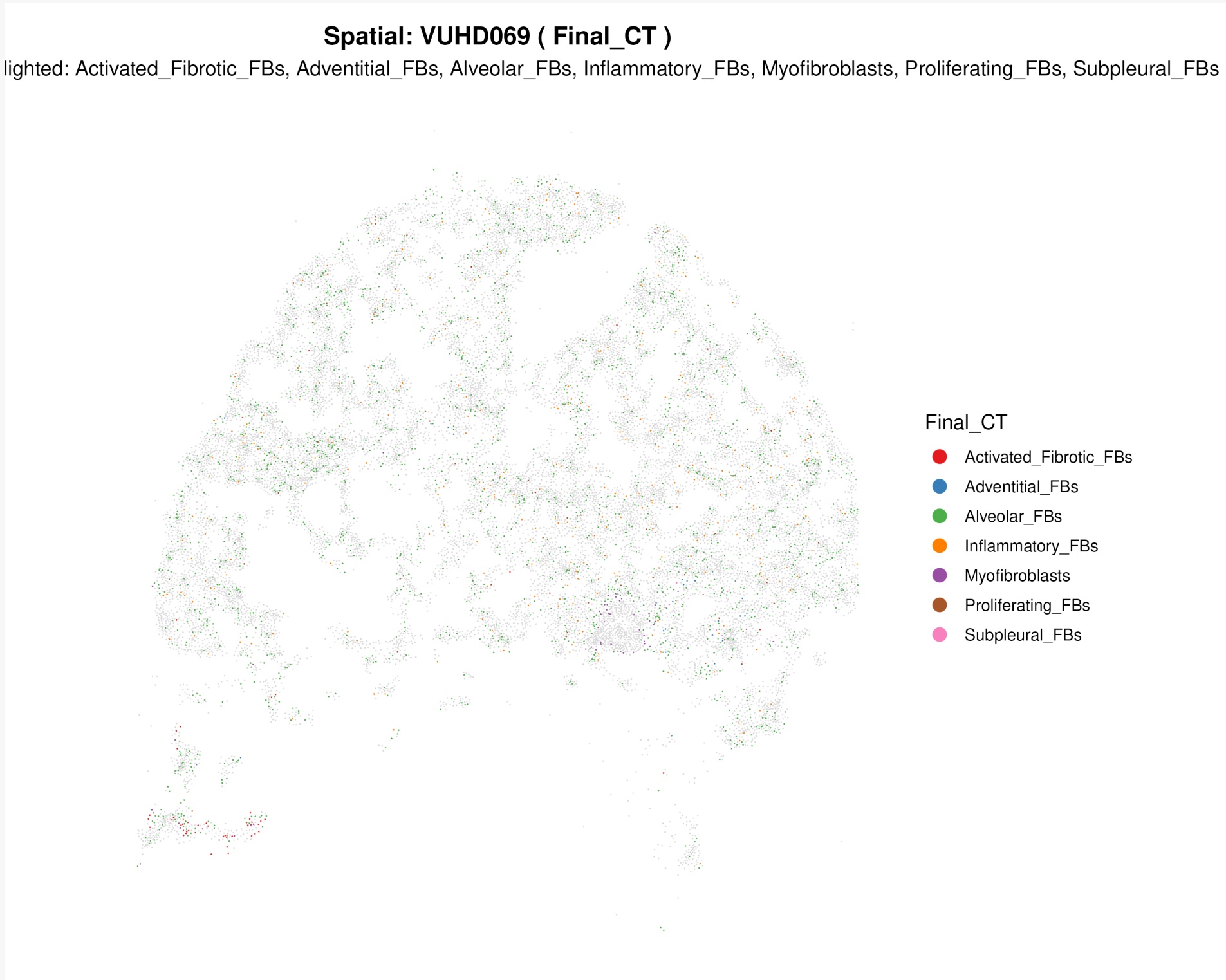

Figure E2: Spatial cell-type distribution in Unaffected IPF tissue (Xenium). UMAP overlay reveals fibroblast subpopulation geography across seven distinct niches.

bioRxiv preprint doi: https://doi.org/10.64898/2026.08.08.743577; this version posted August 14, 2026. The copyright holder for this preprint (which was not certified by peer review) is the author/funder, who has granted bioRxiv a license to display the preprint in perpetuity. It is made available under a CC-BY 4.0 International license.

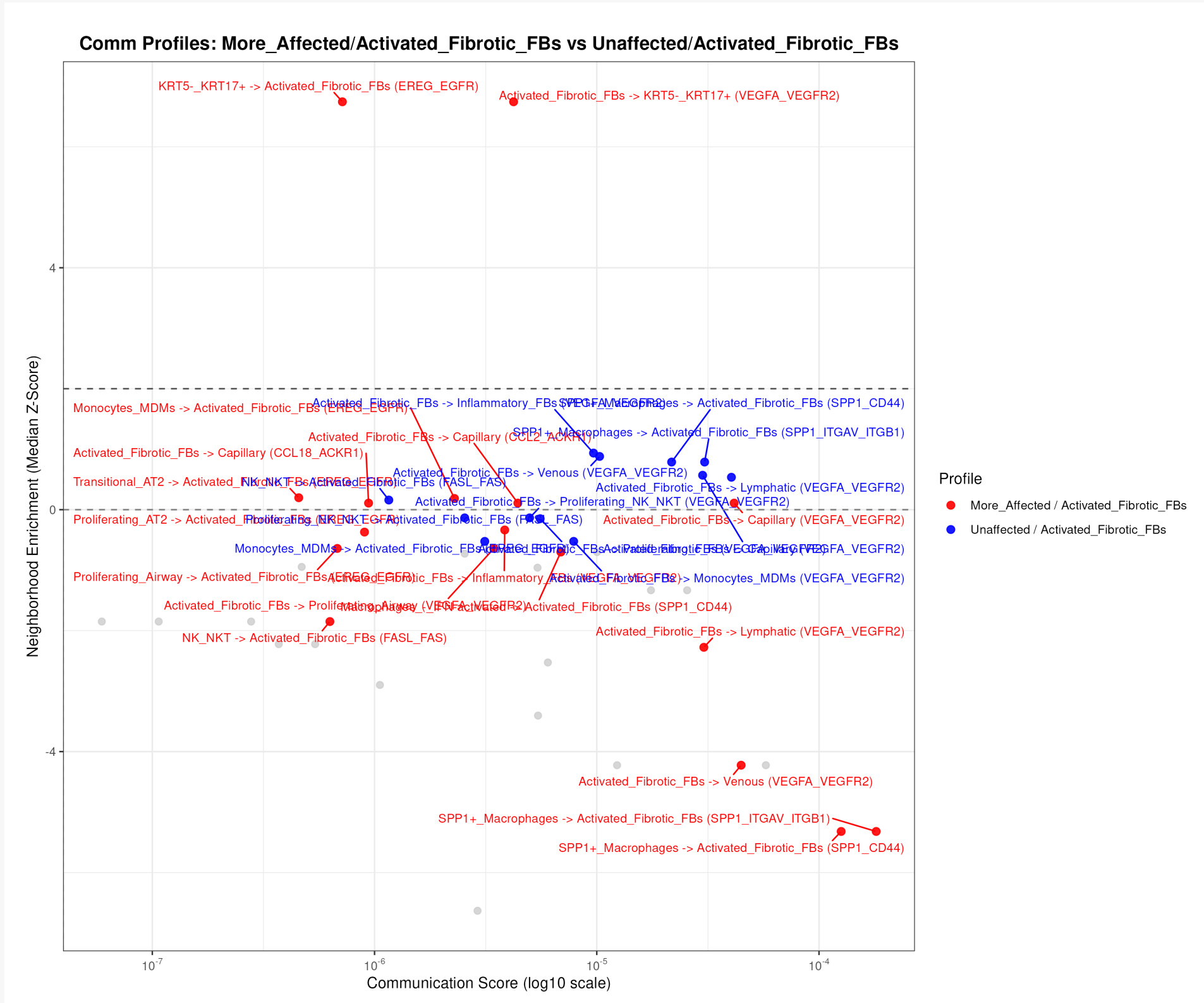

Figure E3: CellChat communication profile for Activated Fibrotic Fibroblasts: More_Affected vs. Unaffected IPF regions. Key gained interactions include SPP1–CD44, VEGFA–VEGFR2, and CCL2–ACKR1 (46 total interactions in More_Affected, 33 unique to the fibrotic state).

Step 5 output: iLINCS drug connectivity. The 3,563-gene alveolar fibroblast IPF signature was screened against **2,383 compounds** in the LINCSCP library (reversal mode). The top 15 candidates ranked by anticorrelation score:

bioRxiv preprint doi: https://doi.org/10.64898/2026.08.08.743577; this version posted August 14, 2026. The copyright holder for this preprint (which was not certified by peer review) is the author/funder, who has granted bioRxiv a license to display the preprint in perpetuity. It is made available under a CC-BY 4.0 International license.

Rank	Drug	Score	Targets	Mechanism
1	**Saracatinib**	**−0.451**	ABL1, SRC	Src/Abl dual kinase inhibitor
2	TWS 119	−0.393	GSK3B	GSK-3β inhibitor (Wnt activator)
3	Tivozanib	−0.392	VEGFRs, PDGFRs	Multi-VEGFR/PDGFR TKI
4	LY2874455	−0.389	FGFR1–4	Pan-FGFR inhibitor
5	Linifanib	−0.386	CSF1R, FLTs, PDGFRs	Multi-RTK inhibitor
6	CI-1040	−0.385	MAP2K1/2	MEK1/2 inhibitor
7	Tozasertib	−0.385	ABL1, AURKs, JAK2	Aurora/Abl kinase inhibitor
8	Gefitinib	−0.383	EGFR	EGFR TKI
9	WH-4-025	−0.382	—	—
10	Tamatinib	−0.380	SYK	SYK kinase inhibitor
11	Fedratinib	−0.377	FLT3, JAK2	JAK2/FLT3 inhibitor
12	BX-795	−0.374	PDK1	PDK1 inhibitor
13	Canertinib	−0.370	EGFR, ERBB2/4	Pan-ErbB inhibitor
14	Pazopanib	−0.365	Multi-RTK	Broad-spectrum TKI
15	LDN 193189	−0.362	ACVR1, BMPR1A	BMP/ALK2 inhibitor

Mechanistic convergence: The top hits cluster around Src/Abl kinase, VEGFR/PDGFR signaling, FGFR, MEK/ERK, and JAK2, all pathways directly implicated in the enriched fibroblast program (cell adhesion, migration, ECM remodeling, HIF-1 signaling). Saracatinib was autonomously selected as the priority candidate for downstream DART analysis.

Steps 6–7 output: DART cell-type impact analysis. The supervisor agent retrieved Saracatinib's own iLINCS compound perturbation signature and executed DART, correlating this signature against **475 lung cell-type/disease signatures** across HLCA, BPD_Sun, and HCA_fetal datasets.

bioRxiv preprint doi: https://doi.org/10.64898/2026.08.08.743577; this version posted August 14, 2026. The copyright holder for this preprint (which was not certified by peer review) is the author/funder, who has granted bioRxiv a license to display the preprint in perpetuity. It is made available under a CC-BY 4.0 International license.

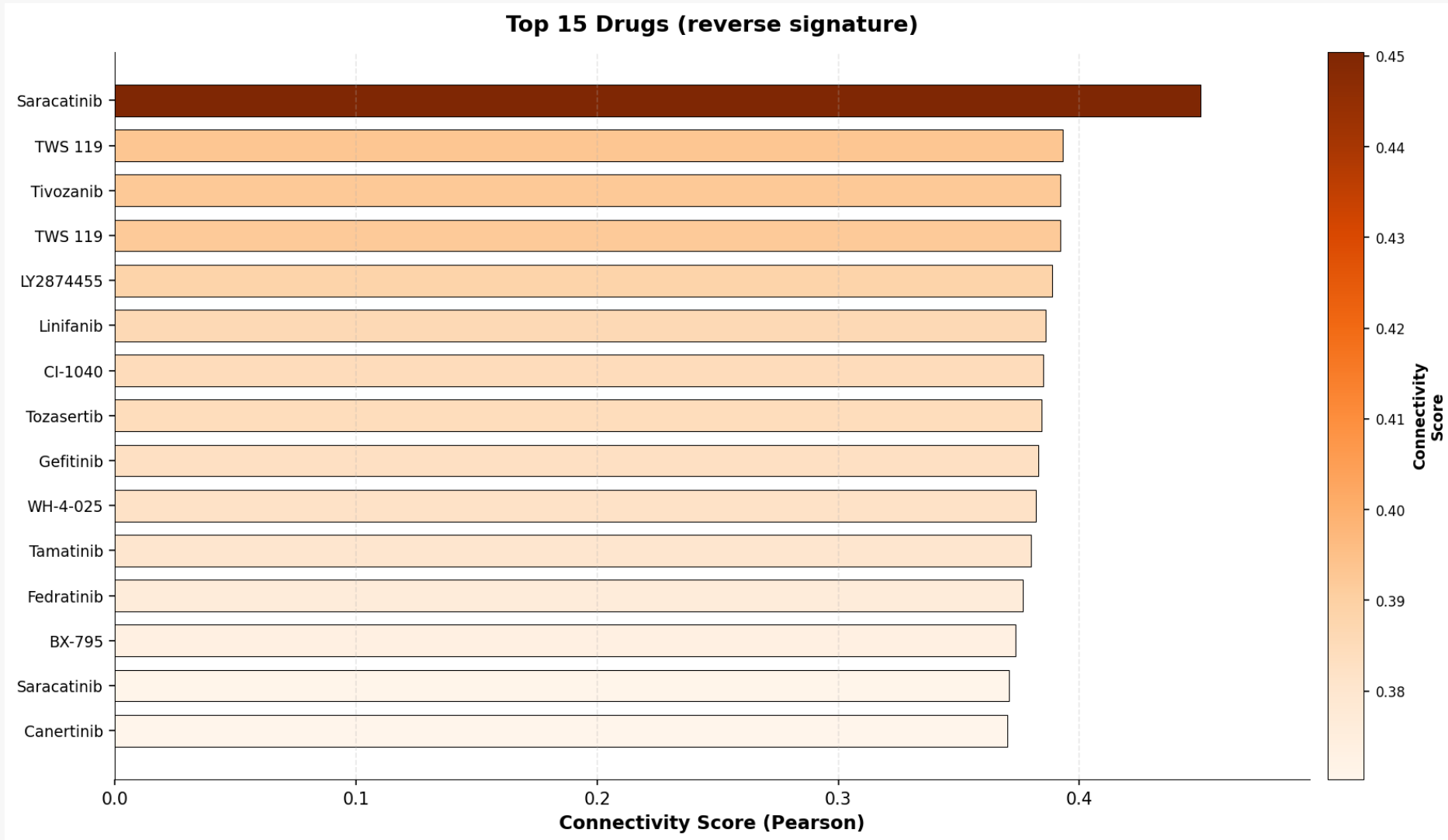

Figure E4: iLINCS drug connectivity screen for the IPF alveolar fibroblast signature (reversal mode, 2,383 compounds). Saracatinib (Src/Abl inhibitor) achieves the highest anticorrelation score ($r = -0.451$), followed by VEGFR/FGFR/MEK inhibitors, consistent with the enriched fibroblast transcriptional program.

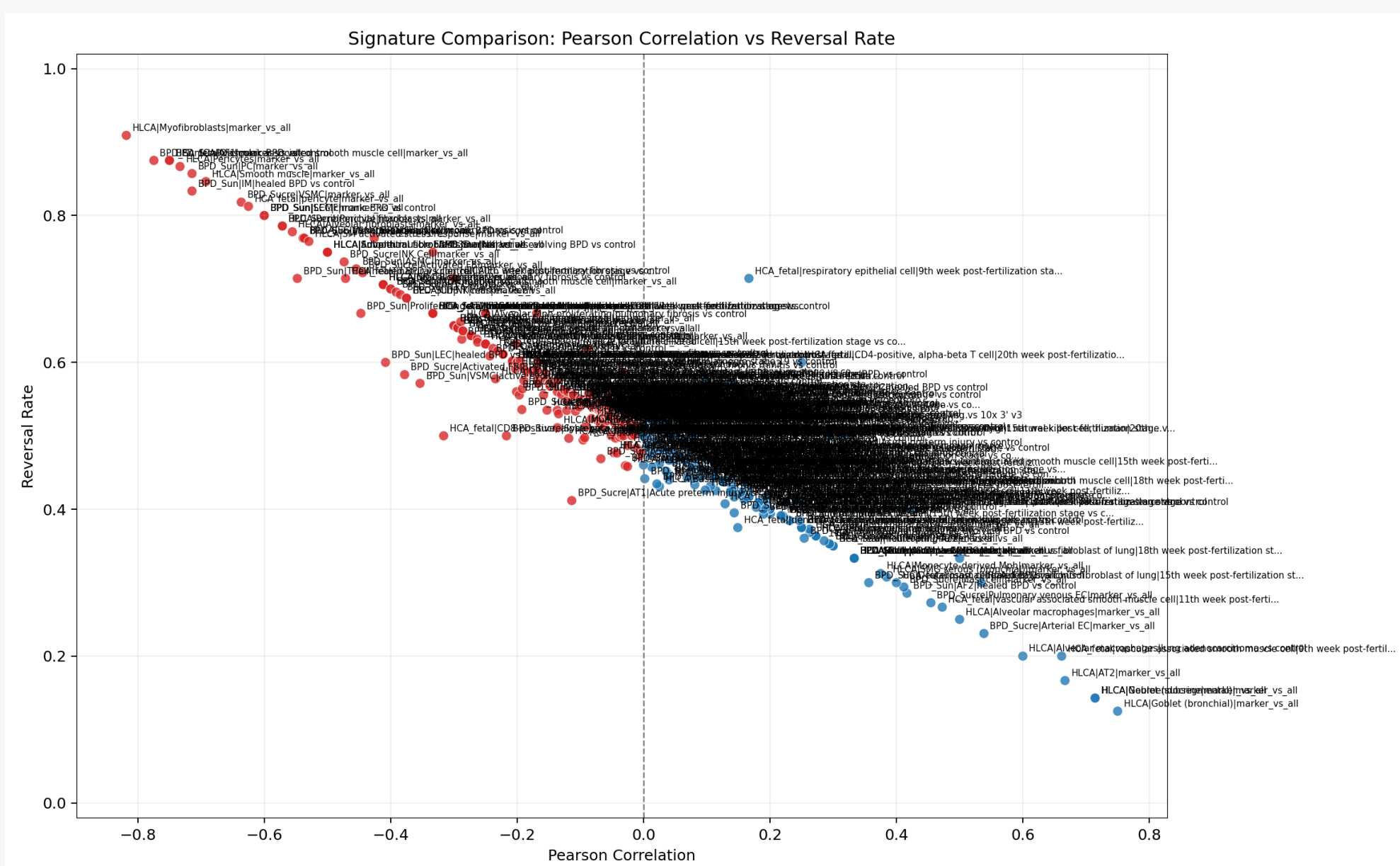

Figure E5: DART cell-type impact analysis for Saracatinib across 475 lung signatures. Negative Pearson r (blue) indicates therapeutic reversal of a signature; positive r (red) indicates mimicry. Saracatinib achieves strong reversal of myofibroblast ($r = -0.818$), smooth muscle, and pericyte marker programs, with a favorable safety profile in alveolar epithelial cells.

Strongest Reversal Signatures (On-Target Therapeutic Benefit):

bioRxiv preprint doi: https://doi.org/10.64898/2026.08.08.743577; this version posted August 14, 2026. The copyright holder for this preprint (which was not certified by peer review) is the author/funder, who has granted bioRxiv a license to display the preprint in perpetuity. It is made available under a CC-BY 4.0 International license.

Cell Type	Dataset	Comparison	r	RR	Key Reversed Genes
**Myofibroblasts**	HLCA	marker vs. all	**−0.818**	**90.9%**	COL1A1, TPM1, MYL9, MYLK, MMP2, SERPINE1
CAP2 capillaries	BPD_Sun	BPD vs. ctrl	−0.775	87.5%	KAT6A, NCOA3, COL4A1, SOX4, MEF2C
AF1 fibroblasts	BPD_Sun	marker vs. all	−0.750	87.5%	MACF1, MYLK, MYO10, TBX2, GLI2
Vascular SMC	HCA_fetal	marker vs. all	−0.750	87.5%	MYL9, TPM1, COL4A1, COL1A1, MYLK
Pericytes	HLCA	marker vs. all	−0.733	86.7%	COL4A1, TBX2, TPM1, MYL9, MYLK
Smooth muscle	HLCA	marker vs. all	−0.692	84.6%	MYL9, TPM1, MYLK, COL4A1, COL1A1
Peribronchial FBs	HLCA	marker vs. all	−0.571	78.6%	COL1A1, MMP2, MYL9, S100A13, P4HA2
Alveolar FBs	HLCA	marker vs. all	−0.556	77.8%	MYLK, MMP2, TBX2, COL1A1, CSRP1

The reversal signature is dominated by contractile/ECM genes (COL1A1, MYL9, MYLK, TPM1, MMP2, SERPINE1), the core fibrotic effector program, across multiple mesenchymal and vascular cell types.

IPF-Specific Reversal Across Cell Types (HLCA disease signatures):

Cell Type	Pearson r	Reversal Rate
EC general capillary	−0.537	76.9%
Pre-TB secretory	−0.412	70.6%
EC arterial	−0.300	71.4%
Alveolar Mph proliferating	−0.289	65.5%
NK cells	−0.263	62.8%
CD8 T cells	−0.234	57.8%
Alveolar fibroblasts	−0.207	60.2%

Saracatinib's reversal effect extends beyond fibroblasts to vascular, immune, and epithelial compartments in IPF, consistent with Src kinase's broad role in cell signaling.

Step 8 output: safety profile. DART identified positive correlations between Saracatinib's perturbation signature and normal cell-type identity programs, indicating potential off-target effects:

Cell Type	r	Concern
Goblet (bronchial)	+0.750	Mimics goblet identity genes (XBP1, SPDEF, MUC1); potential mucus secretion disruption
Goblet (subsegmental)	+0.714	Same mucus program concern
Neuroendocrine	+0.714	Mimics neuroendocrine markers (SNAP25, STMN1); monitor for neuroendocrine effects
AT2 cells	+0.667	Mimics AT2 identity (FGFR2); potential surfactant biology perturbation
Alveolar macrophages	+0.500	Mimics macrophage markers (PPARG, APOE); immune surveillance impact

bioRxiv preprint doi: https://doi.org/10.64898/2026.08.08.743577; this version posted August 14, 2026. The copyright holder for this preprint (which was not certified by peer review) is the author/funder, who has granted bioRxiv a license to display the preprint in perpetuity. It is made available under a CC-BY 4.0 International license.

Interpretation. Saracatinib exhibits strong reversal of fibrotic mesenchymal programs, particularly in myofibroblasts ($r = -0.818$, 90.9% reversal) and perivascular populations, with the effect extending across vascular and immune compartments, indicating system-level remodeling of the fibrotic niche. The positive correlations with goblet and AT2 cells indicate that Saracatinib's signature overlaps with normal epithelial identity programs; this does not necessarily indicate toxicity but warrants monitoring of mucociliary function and surfactant biology in preclinical models. LungChat prioritized Saracatinib through iLINCS drug-connectivity screening and then used DART to evaluate its cell-type effects. The compound has been evaluated in the STOP-IPF Phase 1b/2a clinical trial for IPF (NCT04598919), providing external support for the biological relevance of the overall prioritization.

This entire analysis was completed from a single user prompt in a fresh session with no prior conversation history, demonstrating LungChat's capacity for autonomous, zero-shot therapeutic discovery with complete provenance.

E.2 COPD: Fluticasone Propionate Discovery

Supplementary E2. COPD DART Case Study: Cell-Type-Resolved Zero-Shot Therapeutic Discovery

User prompt (verbatim).

> *Identify therapeutic targets and drug repurposing candidates for COPD, separately considering each cell type. Among the top 10 hits for each cell type, which is the most promising therapeutic given prior clinical data for COPD in which cell type. For this drug, show me the top dysregulated genes in that cell population then evaluate its cell-type-specific impact across all lung populations in adults and in utero, to determine on-target efficacy and off-target safety risks.*

Supervisor decomposition. LungChat's supervisor agent decomposed this single natural-language prompt into a four-stage pipeline with extensive parallelization across cell types:

1. **Per-cell-type DEG signatures:** Generate COPD vs. control DEGs independently for each of the 14 lung cell types in HLCA
2. **Per-cell-type iLINCS screening:** Run drug connectivity analysis on each cell type's signature in parallel, producing independent top-10 drug lists per population
3. **Clinical evidence cross-referencing:** Scan all top-10 lists for drugs with documented COPD clinical trial history; select the most clinically validated candidate
4. **DART cell-type impact:** For the selected drug, generate a volcano plot of DEGs in its primary cell type; then execute DART across adult (HLCA) and fetal (HCA_fetal) lung populations

Unlike the IPF case study, this workflow independently screens **14 cell types**, enabling cell-type-resolved drug discovery and clinical evidence cross-validation across populations.

bioRxiv preprint doi: https://doi.org/10.64898/2026.08.08.743577; this version posted August 14, 2026. The copyright holder for this preprint (which was not certified by peer review) is the author/funder, who has granted bioRxiv a license to display the preprint in perpetuity. It is made available under a CC-BY 4.0 International license.

Complete execution trace.

Step	Agent	Tool(s)	Operation
1	Supervisor	`decompose`	Parses query; identifies 14 cell types with COPD samples in HLCA; plans parallel iLINCS dispatch
2	Single-Cell	`generate_stats` (×14)	Differential expression for each of 14 cell types (COPD vs. control, HLCA). 8 cell types yield signatures with sufficient gene counts for iLINCS
3	Research	`drug_connectivity_tool` (×8, batched)	iLINCS drug connectivity analysis on each cell type's DEG signature independently (reversal mode, LINCSCP). Returns per-cell-type top-10 ranked drug lists
4	Research	`literature_search_tool`, `clinical_trials_search_tool`	Cross-references all top-10 hits against COPD clinical trial databases. Identifies Fluticasone Propionate (>200 trials, Phase 4 approved) in DC2 and Non-classical Monocyte lists
5	Single-Cell	`plot_differential_expression_volcano`	Volcano plot of 521 DEGs in DC2 cells (COPD vs. control)
6	Supervisor	`query_pseudobulk_expression_data_tool (drug_cell_type_impact)`	Executes DART: correlates Fluticasone Propionate iLINCS signature (978 genes) against 252 lung signatures across HLCA (adult) and HCA_fetal (in utero) datasets
7	Supervisor	Synthesis	Aggregates all outputs into integrated therapeutic report with on-target efficacy and fetal off-target safety profiling

bioRxiv preprint doi: https://doi.org/10.64898/2026.08.08.743577; this version posted August 14, 2026. The copyright holder for this preprint (which was not certified by peer review) is the author/funder, who has granted bioRxiv a license to display the preprint in perpetuity. It is made available under a CC-BY 4.0 International license.

Step 2–3 output: per-cell-type drug screening. Of 14 cell types with COPD DEG signatures, 8 yielded sufficient gene counts for iLINCS analysis. Independent screening produced cell-type-resolved drug rankings:

**Cell Type**	**Top Hits (selected)**	**Clinically Relevant**	**Score Range**
Alv. Macrophages	Manumycin-A, L-Sulforaphane	—	−0.31 to −0.37
AT2	Diphenylpyraline, Camptothecin	Sorafenib (#9), Bosutinib (#10)	−0.61 to −0.69
CD4 T cells	Emodic acid, Cerulenin	Tretinoin (#8)	−0.60 to −0.63
CD8 T cells	MD-041, Rita	Valdecoxib (#7)	−0.49 to −0.55
Classical Mono.	AZD-8330, 10-DEBC	Pimasertib (#7), Rimantadine (#8)	−0.40 to −0.45
**DC2**	Veliparib (#1), Piplartine	**Fluticasone Prop. (#6)**, Erlotinib (#9)	−0.62 to −0.68
NK cells	ML175, CAY10618	Momelotinib (#3)	−0.66 to −0.74
Non-cl. Mono.	Veliparib (#1), Piplartine	**Fluticasone Prop. (#6)**	−0.62 to −0.68

bioRxiv preprint doi: https://doi.org/10.64898/2026.08.08.743577; this version posted August 14, 2026. The copyright holder for this preprint (which was not certified by peer review) is the author/funder, who has granted bioRxiv a license to display the preprint in perpetuity. It is made available under a CC-BY 4.0 International license.

Step 4 output: clinical evidence cross-referencing. Scanning all 80 candidate drugs (top 10 × 8 cell types) against COPD clinical trial databases:

Drug	Cell Types (rank)	COPD Clinical Evidence
**Fluticasone Propionate**	DC2 (#6), Non-cl. Mono. (#6)	**Approved.** >200 COPD trials. Phase 4 completed.
Tretinoin	CD4 T cells (#8)	Phase 2 only (emphysema subtype). Not approved.
Rimantadine	Classical Mono. (#8)	One trial (viral prophylaxis context only).
Others	Various	No COPD trials identified.

Fluticasone Propionate was selected as the priority candidate: the only drug in any top-10 list with Phase 4 COPD approval, emerging independently from both DC2 and Non-classical Monocyte signatures ($p = 3.4 \times 10^{-5}$).

Step 5 output: DC2 differential expression (COPD vs. control). Analysis of DC2 (type 2 dendritic) cells identified **521 DEGs** (COPD vs. control). The top dysregulated genes reveal a shift toward a mature, antigen-presenting phenotype with loss of innate alarm signaling:

Dir.	Gene	LogFC	FDR	Functional Role
↑	CD207	+2.49	9.95×10^{-5}	DC maturation / antigen capture
↑	PKP2	+2.16	6.22×10^{-7}	Desmosomal signaling
↑	MT2A	+1.77	1.13×10^{-5}	Metallothionein, oxidative stress
↑	F2RL2	+1.69	2.86×10^{-4}	Protease-activated receptor
↑	CXCL9	+1.64	4.13×10^{-4}	T cell recruitment chemokine
↓	S100A9	−1.74	4.42×10^{-5}	Alarmin / innate immune activation
↓	CXCL8	−1.73	1.50×10^{-4}	Neutrophil recruitment (IL-8)
↓	MMP14	−1.55	5.58×10^{-4}	Matrix metalloprotease
↓	SIRPA	−1.25	2.64×10^{-6}	Phagocytosis checkpoint
↓	TNFAIP8	−1.20	6.57×10^{-6}	NF-κB / apoptosis regulator
↓	HIF1A	−1.05	1.28×10^{-5}	Hypoxia response
↓	BCL2A1	−1.03	3.29×10^{-4}	Anti-apoptotic

bioRxiv preprint doi: https://doi.org/10.64898/2026.08.08.743577; this version posted August 14, 2026. The copyright holder for this preprint (which was not certified by peer review) is the author/funder, who has granted bioRxiv a license to display the preprint in perpetuity. It is made available under a CC-BY 4.0 International license.

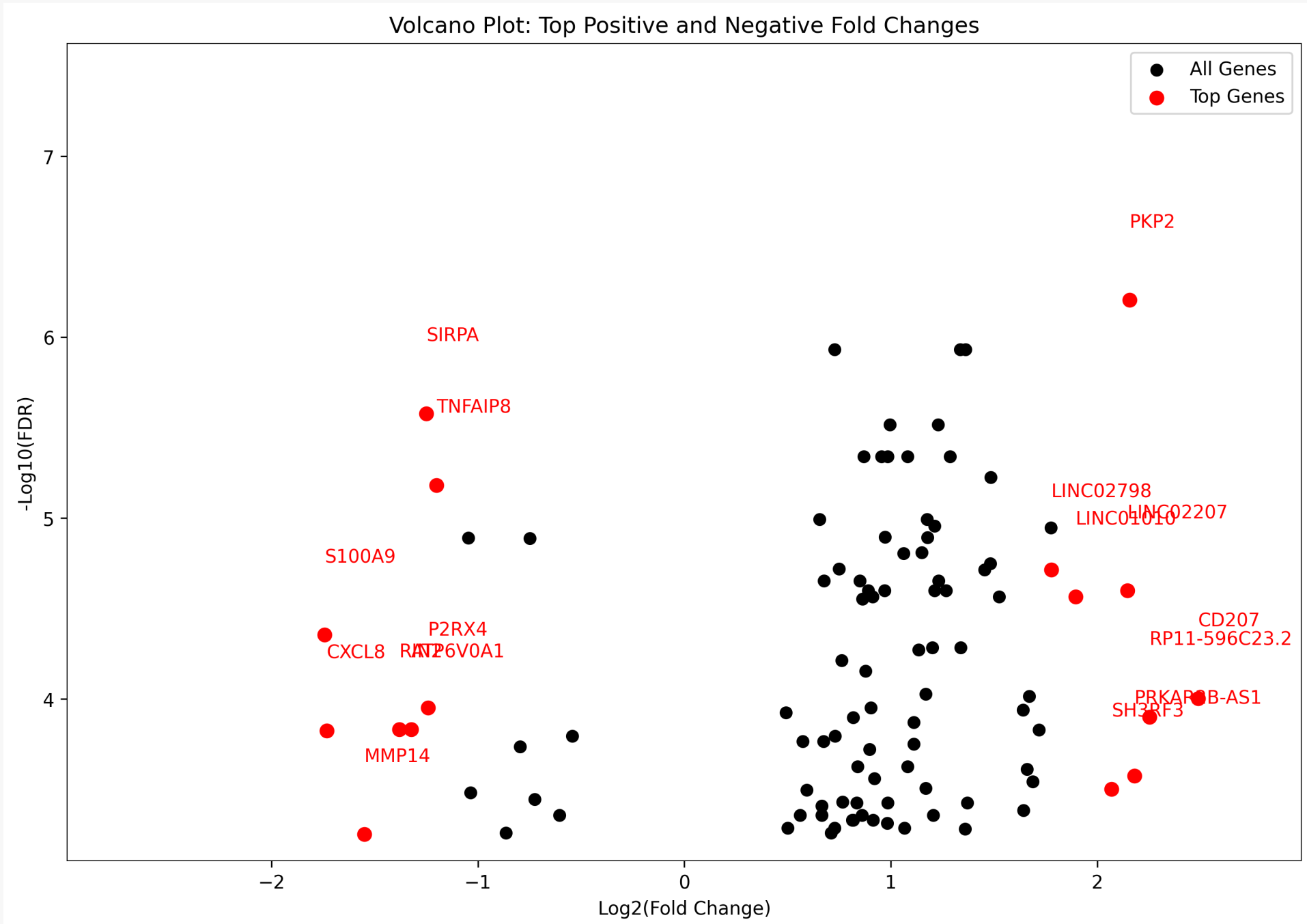

Figure E6: Volcano plot of differential gene expression in COPD vs. control DC2 cells (521 DEGs). Upregulated genes (CD207, CXCL9, PKP2) indicate enhanced antigen presentation; downregulated genes (S100A9, CXCL8, MMP14, HIF1A) indicate loss of innate alarm signaling and hypoxia response.

Step 6 output: DART cell-type impact analysis. The Fluticasone Propionate iLINCS perturbation signature (**978 genes**) was correlated against **252 lung signatures** across HLCA (adult) and HCA_fetal (in utero) datasets. A 100-gene subset was used for impact analysis.

bioRxiv preprint doi: https://doi.org/10.64898/2026.08.08.743577; this version posted August 14, 2026. The copyright holder for this preprint (which was not certified by peer review) is the author/funder, who has granted bioRxiv a license to display the preprint in perpetuity. It is made available under a CC-BY 4.0 International license.

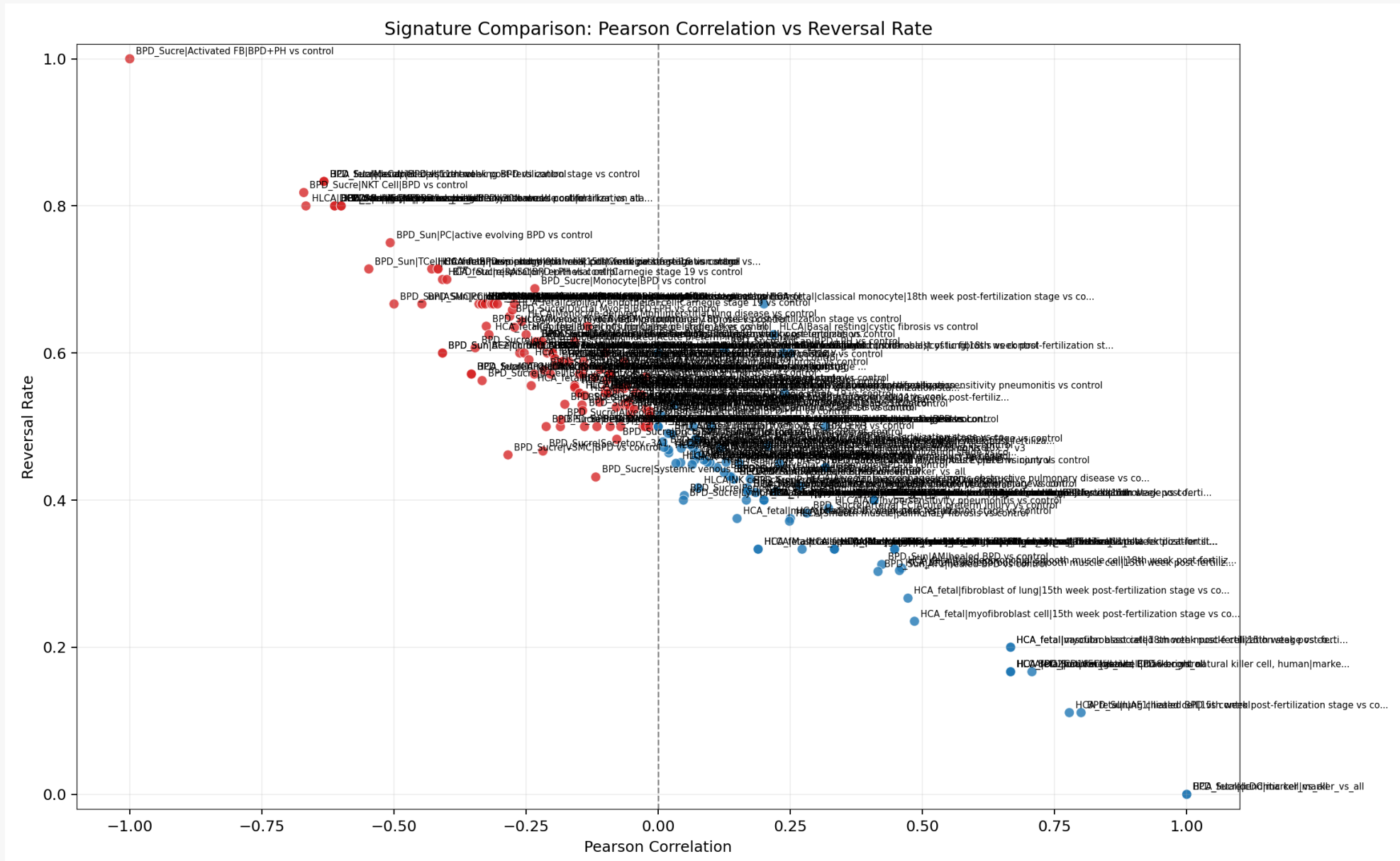

Figure E7: DART cell-type impact analysis for Fluticasone Propionate across 252 lung signatures (HLCA adult + HCA_fetal). Negative Pearson r (blue) indicates therapeutic reversal; positive r (red) indicates signature amplification. DC2 COPD signature shows strong reversal ($r = -0.667$, 80% RR), while fetal dendritic and ciliated cell developmental programs show positive correlation, indicating potential off-target effects during gestation.

bioRxiv preprint doi: https://doi.org/10.64898/2026.08.08.743577; this version posted August 14, 2026. The copyright holder for this preprint (which was not certified by peer review) is the author/funder, who has granted bioRxiv a license to display the preprint in perpetuity. It is made available under a CC-BY 4.0 International license.

On-Target Efficacy (Adult HLCA, COPD Signatures):

Cell Type	Pearson r	RR	Overlap	Key Reversed Genes
**DC2 (COPD vs. ctrl)**	**−0.667**	**80%**	5	JUN↓, USP7↓, CCNH↓, MEF2C↓

The DC2 COPD signature is the only adult HLCA COPD signature with sufficient overlap, and it shows strong anticorrelation ($r = -0.667$, reversal rate 80%), confirming Fluticasone's predicted on-target activity in the cell type where it was identified.

Off-Target Safety (Fetal HCA_fetal, Developmental Programs):

Cell Type	r	RR	Overlap	Safety Interpretation
Dendritic cell (marker)	+1.000	0%	5	Drug matches fetal DC identity genes (PAK1, SYK, MEF2C); risk of disrupting fetal DC development
Ciliated cell (15 wk)	+0.778	11%	9	Drug amplifies ciliated cell developmental program; potential for altered airway epithelial maturation
Myofibroblast (18 wk)	+0.667	20%	5	Overlap with mesenchymal developmental genes (BMP4, CSRP1); possible off-target on lung structural development
Vascular SMC (15 wk)	+0.667	20%	5	Shared signature with vascular developmental program (COL1A1, MEF2C)

Interpretation. Fluticasone Propionate demonstrates strong reversal of the COPD-associated transcriptional program in DC2 dendritic cells ($r = -0.667$, 80% reversal rate), the primary cell type where it was computationally identified. The reversed genes (JUN, MEF2C, CCNH, USP7) are consistent with glucocorticoid receptor (NR3C1)-mediated suppression of inflammatory signaling, mechanistically linking the drug's known anti-inflammatory activity to the COPD DC2 disease program.

The fetal safety analysis reveals a clinically interpretable risk: Fluticasone's signature positively correlates with normal developmental programs in dendritic cells ($r = +1.0$), ciliated epithelial cells ($r = +0.778$), myofibroblasts ($r = +0.667$), and vascular smooth muscle ($r = +0.667$) at 15–18 weeks gestation. This transcriptomic signal is consistent with the need for caution and pregnancy/lactation risk assessment in current drug-labeling frameworks, providing cell-type-resolved molecular evidence for potential effects during fetal airway and mesenchymal development.

The finding that an approved, Phase 4-validated COPD corticosteroid is autonomously identified as the top candidate from cell-type-resolved computational screening, with mechanistically coherent on-target and off-target profiles, provides strong external validation of the DART method's capacity for clinically meaningful drug repurposing. The overlap counts are small (5–9 genes per comparison), so these correlations should be interpreted as directional signals rather than definitive predictions.

This entire analysis, including per-cell-type drug screening across 14 populations, clinical evidence cross-referencing, DC2 differential expression, and dual-dataset (adult + fetal) DART safety profiling,

bioRxiv preprint doi: https://doi.org/10.64898/2026.08.08.743577; this version posted August 14, 2026. The copyright holder for this preprint (which was not certified by peer review) is the author/funder, who has granted bioRxiv a license to display the preprint in perpetuity. It is made available under a CC-BY 4.0 International license.

was completed from a single user prompt in a fresh session with no prior conversation history.

F Architecture ablation details

This appendix provides the full specification and detailed results for the architecture ablation described in Section 2.2.

F.1 Ablation configuration details

Table F1 details the four architecture configurations evaluated. The key design principle: flat-agent baselines preserve all domain knowledge from specialist prompts, ensuring that observed differences reflect architectural choices rather than information asymmetry.

Table F1: Ablation arm specifications. All configurations use the same foundation models, datasets, and evaluation rubrics.

Config	Agent(s)	Prompt	Tool Access	What It Isolates
MA+Sel (production)	5 agents (Supervisor + 4 specialists)	Specialized per agent	Supervisor routing + hybrid selector	Full system baseline
MA−Sel	5 agents (Supervisor + 4 specialists)	Specialized per agent	Supervisor routing, no selector	Selector contribution
Flat+Sel	1 agent	Merged flat prompt	All 51 tools + hybrid selector	Value of hierarchy when tool narrowing retained
Flat−Sel	1 agent	Merged flat prompt	All 51 tools, no selector	Hierarchy + tool narrowing combined

F.2 Context engineering rationale

The multi-agent architecture is motivated by a context-engineering principle: each specialist agent operates in a scoped context window containing only its domain-relevant tools and instructions, preventing cross-domain parameter leakage and instruction dilution. Token efficiency is a measurable consequence of this design (cumulative prompt tokens scale sub-linearly with task complexity), but the primary benefit is preserved reasoning quality under increasing tool and domain complexity.

Table F2 summarizes the specific context problems addressed by the hierarchical design.

Table F2: Context problems addressed by multi-agent hierarchy vs. flat architecture.

Problem	Flat Agent	Multi-Agent
Tool confusion	51 tool schemas compete for attention	Sub-agent sees $\sim$13 domain-relevant tools
Cross-domain contamination	HLCA metadata fields leak into spatial parameters	Each domain operates in isolated context
Instruction dilution	Combined prompts across all domains	Each sub-agent receives 13–29KB scoped instructions
Error containment	Bad tool call poisons entire reasoning chain	Sub-agent fails locally; supervisor retries or degrades
Scalability	Adding tools increases token overhead for ALL queries	New tools affect only their specialist agent
Tool attention bloat	Agent overly eager to invoke *some* tool	Routing layer abstracts tools from supervisor

The context-partitioning principles underlying our design align with several recent findings. Liu et al. [15] demonstrated systematic "lost in the middle" degradation where LLM accuracy drops for mid-context information, a phenomenon exacerbated by monolithic context accumulation. Anthropic [2] identifies sub-agent delegation and context compaction as primary mitigations for context degradation in long-running agents. Zhang et al. [30] showed that multi-agent collaboration through scoped worker contexts outperforms single-agent systems by up to 10% on long-context tasks.

bioRxiv preprint doi: https://doi.org/10.64898/2026.08.08.743577; this version posted August 14, 2026. The copyright holder for this preprint (which was not certified by peer review) is the author/funder, who has granted bioRxiv a license to display the preprint in perpetuity. It is made available under a CC-BY 4.0 International license.

F.3 Full single-step ablation results

Table F3 provides the complete per-metric breakdown for all 50 single-step queries across 4 ablation modes (n=3 repetitions each, 600 total evaluations).

Table F3: Full single-step ablation results (50 queries × 3 reps × 4 modes = 600 evaluations). **Bold** indicates best per metric. Quality: higher is better. Efficiency: lower is better.

	Metric	MA+Sel	MA−Sel	Flat+Sel	Flat−Sel
Quality	LLM Judge	0.893 ± 0.31	0.927 ± 0.26	0.947 ± 0.23	**0.980** ± 0.14
	Tool Selection	0.920 ± 0.27	0.933 ± 0.25	0.927 ± 0.24	**0.980** ± 0.14
	Config Match	0.927 ± 0.26	0.940 ± 0.24	0.913 ± 0.28	**0.947** ± 0.23
	Items Coverage	0.891 ± 0.27	0.919 ± 0.23	**0.927** ± 0.22	0.924 ± 0.21
	Execution Success	0.973 ± 0.16	**0.980** ± 0.14	0.973 ± 0.16	0.973 ± 0.16
	Metadata Consistency	**1.000** ± 0.00	0.973 ± 0.16	0.973 ± 0.16	**1.000** ± 0.00
Efficiency	Mean Latency (s)	50.2	56.7	46.3	**39.5**
	Mean Tokens (K)	**197**	212	330	401
	Mean Tool Calls	**2.05**	2.18	3.79	3.37

Observations.

1. **Quality**: Flat−Sel achieves the highest LLM Judge score (0.980) and Tool Selection accuracy (0.980), but the absolute gap to the production configuration (MA+Sel) is ≤0.09 across all metrics.
2. **Token efficiency**: MA+Sel consumes ∼2× fewer tokens than Flat−Sel (197K vs. 401K), reflecting context partitioning across specialist agents.
3. **Latency**: Flat−Sel is fastest (39.5s) because it avoids supervisor→sub-agent routing overhead, while MA+Sel adds ∼10s for delegation.
4. **Tool calls**: MA+Sel averages 2.05 tool calls vs. Flat−Sel's 3.37, because flat agents attempt more exploratory tool calls without hierarchical guidance.
5. **Metadata consistency**: MA+Sel achieves perfect metadata consistency (1.000), suggesting that scoped contexts improve parameter grounding for dataset-specific metadata fields.

F.4 Security and grounded abstention results

Security (13 queries × 3 reps × 4 modes = 156 evaluations). All four architectures achieved 100% prevention of explicit tool execution and secret leakage under prompt injection, demonstrating that core supervisor prompt engineering and sandbox boundaries are robustly engineered regardless of tool configuration. MA+Sel showed slightly lower refusal coherence (82.1% vs. 92.3%) because the selector mechanism occasionally attempts to legitimately route complex adversarial language before recognizing hostile intent.

Grounded abstention (17 queries × 3 reps × 4 modes = 204 evaluations). This benchmark provides the strongest architectural validation. When presented with out-of-scope queries, flat architectures (whose context windows expose all 51 tool schemas) become overly eager to invoke *some* tool (54.9% disallowed-tool

bioRxiv preprint doi: https://doi.org/10.64898/2026.08.08.743577; this version posted August 14, 2026. The copyright holder for this preprint (which was not certified by peer review) is the author/funder, who has granted bioRxiv a license to display the preprint in perpetuity. It is made available under a CC-BY 4.0 International license.

blocking vs. 90.2% for MA+Sel). This +35% absolute improvement directly validates the context-engineering hypothesis: abstracting domain tools behind a routing layer forces the top-level agent to reason about intent before invoking capabilities, yielding superior grounded refusal. MA+Sel also produces fewer invalid output artifacts (86.3% clean vs. 70.6% for Flat−Sel), indicating that context isolation reduces spurious output generation.

F.5 Multi-tool orchestration results

Based on the monotonic cost-quality ordering in the 4-arm single-step ablation (MA+Sel: best cost, Flat−Sel: best accuracy), the multi-tool and decomposition-stress benchmarks were evaluated on the two boundary configurations: MA+Sel (production) and Flat−Sel.

Quality (10 queries $\times$ 3 reps $\times$ 2 modes $=$ 60 evaluations). Table F4 summarizes multi-tool orchestration results. Both architectures achieve identical strict pass rates (66.7%) and comparable mean rubric scores (MA+Sel: 3.10/4, Flat−Sel: 3.23/4). Per-question analysis reveals task-dependent architecture preference rather than a global advantage: MA+Sel wins on queries requiring gene regulatory network generation with literature search or multi-dataset cell frequency analysis (MT03, MT04, MT06, MT08), where the supervisor's decomposition into parallel sub-agent tasks produces more coherent synthesis. Flat−Sel wins on queries with broader tool orchestration (MT01, MT05, MT09, MT10), where direct access to all tools avoids routing overhead.

Table F4: Multi-tool orchestration ablation (10 queries $\times$ 3 reps $\times$ 2 modes). Quality: higher is better. Efficiency: lower is better. **Bold** indicates best per metric.

	Metric	MA+Sel	Flat−Sel
Quality	Execution Success	0.967 ± 0.18	$\mathbf{1.000} \pm 0.00$
	Mean Rubric Score (0–4)	3.10 ± 1.51	$\mathbf{3.23} \pm 1.36$
	Strict Pass Rate (%)	66.7	66.7
	Task Coverage (%)	80.0	**83.3**
	Trace Fidelity (%)	76.7	**80.0**
	Cross-Domain Synthesis (%)	73.3	**76.7**
Efficiency	Mean Latency (s)	303.5	**151.7**
	Mean Tokens (K)	**1,477**	1,540
	Mean Tool Calls	**12.0**	18.6
	Hard Failure Rate (%)	10.0	**6.7**

Efficiency. Flat−Sel is 2$\times$ faster (152s vs. 304s) due to the absence of supervisor→sub-agent delegation overhead. However, total token consumption is nearly identical ($\sim$1.5M per query), indicating the latency difference arises from sequential API calls in the delegation chain, not from additional computation. Flat−Sel uses 55% more tool calls (18.6 vs. 12.0), compensating for the lack of hierarchical routing intelligence with

bioRxiv preprint doi: https://doi.org/10.64898/2026.08.08.743577; this version posted August 14, 2026. The copyright holder for this preprint (which was not certified by peer review) is the author/funder, who has granted bioRxiv a license to display the preprint in perpetuity. It is made available under a CC-BY 4.0 International license.

broader exploratory tool invocation. The cost crossover relative to single-step queries is notable: on multi-tool queries, Flat−Sel is modestly cheaper, because each sub-agent invocation in the hierarchical architecture carries context setup overhead.

F.6 Decomposition stress results

Quality (10 queries $\times$ 3 reps $\times$ 2 modes $= 60$ evaluations). Table F5 summarizes decomposition-stress results. Both architectures achieve near-ceiling quality: >83% strict pass rates with zero hard failures and mean rubric scores >5.3/6. This is the highest-quality benchmark in the evaluation suite, demonstrating that LungChat's analytical quality on complex decomposition tasks comes from its domain engineering (curated tools, pre-computed warehouse, expert prompts) rather than from the multi-agent hierarchy.

Table F5: Decomposition stress ablation (10 queries $\times$ 3 reps $\times$ 2 modes). Quality: higher is better. Efficiency: lower is better. **Bold** indicates best per metric.

	Metric	MA+Sel	Flat−Sel
Quality	Mean Rubric Score (0–6)	**5.47** ± 1.45	5.33 ± 1.81
	Strict Pass Rate (%)	83.3	**86.7**
	Hard Failure Rate (%)	**0.0**	**0.0**
	Hypothesis Coverage (%)	**93.3**	86.7
	Evidence for Each Side (%)	**93.3**	86.7
	Epistemic Calibration (%)	90.0	90.0
	Evidence Diversity (%)	90.0	90.0
	Cross-Dataset Integration (%)	**96.7**	90.0
Eff.	Mean Latency (s)	570.2	**214.9**
	Mean Tokens (K)	**1,965**	1,983
	Mean Tool Calls	**21.9**	29.3

Mechanistic observations. MA+Sel shows a modest edge on criteria that specifically reward structured decomposition: hypothesis coverage (+6.6pp), evidence for each side (+6.6pp), and cross-dataset integration (+6.7pp). These criteria reward the supervisor's ability to dispatch independent analyses to specialist agents before synthesis. Flat−Sel matches or exceeds on mechanistic depth (90.0% vs. 83.3%), suggesting that direct tool access without routing intermediation can yield deeper single-branch reasoning. Flat−Sel is 2.7× faster (215s vs. 570s) with dramatically lower variance, making it more predictable for complex queries.

F.7 Combined evaluation summary

Table F6 provides a consolidated view across all five evaluation tracks (100 unique questions, 1,080 total evaluations). No single configuration dominates all dimensions.

bioRxiv preprint doi: https://doi.org/10.64898/2026.08.08.743577; this version posted August 14, 2026. The copyright holder for this preprint (which was not certified by peer review) is the author/funder, who has granted bioRxiv a license to display the preprint in perpetuity. It is made available under a CC-BY 4.0 International license.

Table F6: Combined evaluation summary across all five tracks. **Bold** indicates the better configuration per metric. Quality metrics are reported as percentages; token counts are in thousands.

**Track (N)**	**Key Metric**	**MA+Sel**	**Flat−Sel**	**Winner**
Single-Step (600)	LLM Judge (%)	89.3	**98.0**	Flat (−8.7pp)
Single-Step (600)	Mean Tokens (K)	**197**	401	MA (2× fewer)
Security (156)	No Leak / No Exec (%)	**100.0**	**100.0**	Tie
Security (156)	Mean Tokens (K)	**38**	89	MA (2.3× fewer)
Grounded Abstention (204)	No Disallowed Tools (%)	**90.2**	54.9	**MA (+35.3pp)**
Grounded Abstention (204)	Mean Tokens (K)	**131**	340	MA (2.6× fewer)
Multi-Tool (60)	Mean Rubric (0–4)	3.10	**3.23**	≈Tie (−0.13)
Multi-Tool (60)	Mean Tokens (K)	**1,477**	1,540	≈Tie
Decomp. Stress (60)	Mean Rubric (0–6)	**5.47**	5.33	≈Tie (+0.14)
Decomp. Stress (60)	Mean Tokens (K)	**1,965**	1,983	≈Tie

The production multi-agent architecture (MA+Sel) achieves the best profile across the Pareto frontier: 89% single-step accuracy (vs. 98% for Flat−Sel), 90% disallowed-tool blocking (vs. 55% for Flat−Sel), comparable multi-tool and decomposition-stress reasoning, and 2× token efficiency on the dominant single-step query class. The flat-agent baseline demonstrates that LungChat's domain knowledge, not its architecture, is the primary driver of analytical quality, while the multi-agent hierarchy provides the safety, cost, and scalability properties required for production deployment.

F.8 Discussion and limitations

Across all five evaluation tracks (100 questions, 1,080 evaluations), analytical quality is architecture-neutral: both multi-agent and flat-agent configurations achieve comparable accuracy on single-step, multi-tool, and decomposition-stress queries. The multi-agent hierarchy's measurable contribution is on dimensions that accuracy-only benchmarks do not capture: a +35pp improvement in grounded abstention (preventing the system from invoking biological tools on out-of-scope queries), 2× token-cost reduction on the dominant single-step query class, and structural guarantees for error containment and extensibility.

The current evaluation focuses on single-turn queries. While the stateless sub-agent design provides structural advantages for multi-turn session continuity, where each sub-agent invocation operates in a fresh context independent of prior turns, we have not empirically evaluated this property across long sessions. At `https://chat.lungmap.net`, researchers routinely conduct sessions spanning 10–20 queries; in a monolithic architecture, accumulated context would approach 4M+ tokens after 10 turns, while the multi-agent supervisor carries compact summaries (∼100K tokens) with each sub-agent receiving a fresh context. Quantifying quality degradation over extended sessions is an important direction for future work.

A natural next step is to distill the current LLM-based tool reranker into a lightweight learned reranker trained on successful traces, while retaining LLM fallback for low-confidence or out-of-distribution cases.
